# Online repetitive transcranial magnetic stimulation during working memory in younger and older adults: A randomized within-subject comparison

**DOI:** 10.1371/journal.pone.0213707

**Published:** 2019-03-22

**Authors:** L. Beynel, S. W. Davis, C. A. Crowell, S. A. Hilbig, W. Lim, D. Nguyen, H. Palmer, A. Brito, A. V. Peterchev, B. Luber, S. H. Lisanby, R. Cabeza, L. G. Appelbaum

**Affiliations:** 1 Department of Psychiatry and Behavioral Science, Duke University School of Medicine, Durham, North Carolina, United States of America; 2 Department of Neurology, Duke University School of Medicine, Durham, North Carolina, United States of America; 3 Center for Cognitive Neuroscience, Duke University, Durham, North Carolina, United States of America; 4 Department of Biomedical Engineering, Duke University, Durham, North Carolina, United States of America; 5 Department of Electrical and Computer Engineering, Duke University, Durham, North Carolina, United States of America; 6 Department of Neurosurgery, Duke University School of Medicine, Durham, North Carolina, United States of America; 7 National Institute of Mental Health, Bethesda, Maryland, United States of America; 8 Department of Psychology & Neuroscience, Duke University, Durham, North Carolina, United States of America; University of Regensburg, GERMANY

## Abstract

Working memory is the ability to perform mental operations on information that is stored in a flexible, limited capacity buffer. The ability to manipulate information in working memory is central to many aspects of human cognition, but also declines with healthy aging. Given the profound importance of such working memory manipulation abilities, there is a concerted effort towards developing approaches to improve them. The current study tested the capacity to enhance working memory manipulation with online repetitive transcranial magnetic stimulation in healthy young and older adults. Online high frequency (5Hz) repetitive transcranial magnetic stimulation was applied over the left dorsolateral prefrontal cortex to test the hypothesis that active repetitive transcranial magnetic stimulation would lead to significant improvements in memory recall accuracy compared to sham stimulation, and that these effects would be most pronounced in working memory manipulation conditions with the highest cognitive demand in both young and older adults. Repetitive transcranial magnetic stimulation was applied while participants were performing a delayed response alphabetization task with three individually-titrated levels of difficulty. The left dorsolateral prefrontal cortex was identified by combining electric field modeling to individualized functional magnetic resonance imaging activation maps and was targeted during the experiment using stereotactic neuronavigation with real-time robotic guidance, allowing optimal coil placement during the stimulation. As no accuracy differences were found between young and older adults, the results from both groups were collapsed. Subsequent analyses revealed that active stimulation significantly increased accuracy relative to sham stimulation, but only for the hardest condition. These results point towards further investigation of repetitive transcranial magnetic stimulation for memory enhancement focusing on high difficulty conditions as those most likely to exhibit benefits.

## Introduction

Working memory (WM) is a cognitive ability that allows the maintenance and manipulation of information that is retained in the mind for brief periods. Through the interface between long-term memories and moment-to-moment information available in the environment, WM allows humans to carry out successful goal-directed behaviors [1]. As such, WM is widely involved in the achievement of complex tasks such as learning, decision making, and reasoning. Critically, decline in WM is a major factor in cognitive impairment that accompanies healthy aging [2,3]. Therefore, many different approaches are being explored as possible interventions to enhance these abilities, including non-invasive brain stimulation methods such as repetitive transcranial magnetic stimulation (rTMS). rTMS uses brief, high intensity magnetic fields to depolarize neurons underneath the magnetic coil and has been shown to alter cortical function in a frequency-dependent manner, with high frequencies (≥ 5Hz) generally increasing excitability [4]. Although high frequency rTMS has been shown to reduce WM impairment associated with psychiatric disorders [5,6], only a few studies investigated the effect of rTMS to ameliorate age-related cognitive decline [7,8], and to our knowledge, none of these studies have attempted to enhance WM. The current study, therefore, aimed to bridge this gap by testing the capacity of rTMS to enhance WM in healthy younger and older adults. The goal of this study was to implement state-of-the-art, individual fMRI-guided rTMS with neuronavigation and robotic coil placement to investigate two specific questions whose answers may lead to adoption of this technique for improving WM. To provide clear articulation of a priori hypotheses, this study was pre-registered on ClinicalTrials.gov (NCT02767323).

The first question asked here was related to the effect of rTMS on the manipulation of information in working memory and the interaction of this effect with the task difficulty. WM is assumed to consist of *maintenance* processes that sustain information in an activated state and *manipulation* processes that operate on the maintained information [9]. Compared to maintenance, manipulation is more dependent on dorsolateral prefrontal cortex (DLPFC) [10] which is one of the brain regions shown to be most impaired by aging [2,3]. The few studies that have used rTMS to enhance WM have focused on the *maintenance* process. For example, these studies found that online rTMS to parietal cortex during verbal [11] and spatial [12, 13] WM tasks shortened reaction times relative to sham stimulation. Yet, no rTMS studies have tried to enhance WM manipulation by stimulating DLPFC. In this regard, it is worth noting that several rTMS studies failed to enhance WM (e.g. [14, 15]). One possible explanation is that the WM task was not sufficiently difficult. In fact, greater rTMS enhancement of performance has been found in a WM maintenance task when more items were in WM [11, 16, 17] and in an object identification task, when images were degraded rather than intact [18]. Based on these studies, we hypothesized that rTMS applied over the DLPFC during a WM manipulation task would significantly enhance performance in hard, but not easy trials. In consideration of the frequency of rTMS stimulation, past studies have shown that online rTMS could induce performance enhancement by entraining endogenous task-related oscillatory dynamics. When applied at alpha frequency, online rTMS has been shown to induce a boost in alpha-power band at the targeted region [19] along with corresponding behavioral performance enhancement [20]. Given the important role of theta oscillations in memory processes [21], and based on previous findings demonstrating that 5Hz rTMS resulted in performance enhancement to WM maintenance [11, 22], 5 Hz was selected as the stimulation frequency in the present study.

The second question was related to the modulatory effects of aging on rTMS effects. Although some studies have shown that the effects of single and paired-pulse TMS largely differ with aging (e.g., [23, 24]), to our knowledge only a few studies investigated the effects of rTMS on healthy aging [7, 8]. Complex age-related changes occur in the physiology of the human brain with aging, probably underlying the declines in cognitive abilities, and hence, rTMS effects are likely to be different with participants of different ages. As such, the second goal of this study was to investigate whether rTMS could be as effective in older adults as in younger adults. To prevent confounds between aging and task difficulty stimuli were titrated for each participant so that performance accuracy was equated across individuals as well as age cohorts. Based on this task design, we hypothesized that rTMS would enhance WM manipulation in both young and older adults.

## Methods

### Participants

**Fig 1:**
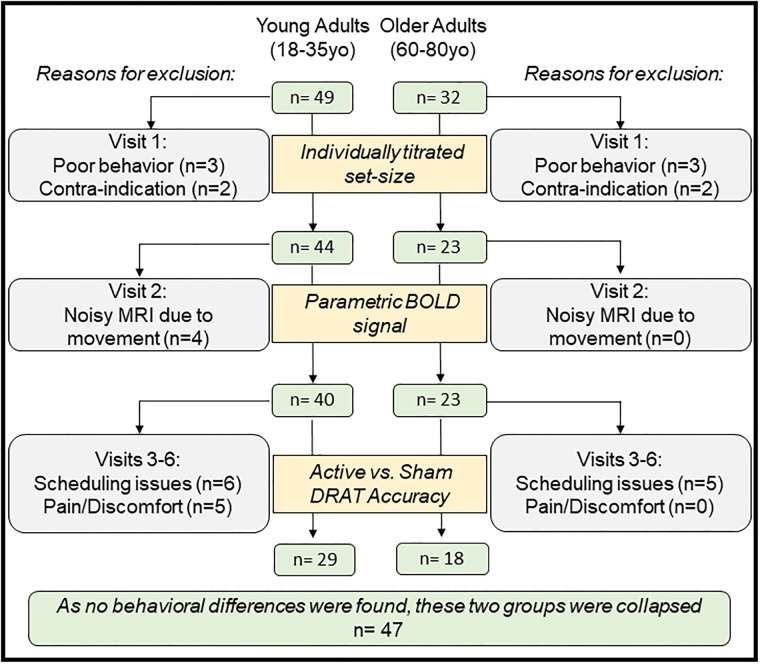
Consort diagram showing the recruitment, exclusion and inclusion numbers.

Fifty-three healthy young adults (18–35 years old) and thirty-two healthy older adults (60–80 years old) were recruited to participate in this single-blind randomized within-subject controlled trial, approved by Duke University Institutional Review Board (IRB protocol #Pro00065334). Recruitment was performed between August 2016 and April 2017 for the young adults; and from January to November 2017 for the older adults. All participants provided written informed consent, approved by Duke University Institutional Review Board (IRB protocol #Pro00065334). Recruitment was performed between August 2016 and April 2017 for the young adults; and from January to November 2017 for the older adults. All participants provided written informed consent for the study, which was approved by Duke University Institutional Review Board (IRB protocol #Pro00065334). The authors confirm that all ongoing and related trials for this intervention are registered on ClinicalTrials.gov (NCT02767323). During the first visit, potential participants were excluded if they had any contraindication to TMS as determined by the TMS Adult Safety Screen [25] (S3 File for the TASS), current or past Axis I psychiatric disorders including substance abuse/dependences or neurological disease as determined by the MINI International Neuropsychiatric Interview, English Version 5.0.0 DSM-IV [26], or a total scaled score < 8 on the Dementia Rating Scale-2 (for the older group only). All participants were also screened for substance use with urine drug screens and women of childbearing potential were screened with urine pregnancy tests. Individuals were excluded if they tested positive in either urine screen (the detailed forms used for inclusion and exclusion criteria used in this study are provided in S4 File). All data were collected in Duke School of Medicine, in the Brain Stimulation Research Center (the adverse effect monitoring plan is provided in S5 File). Sample size was calculated to attain overall alpha significance of 0.05 using two-tailed test on task performance as measured by accuracy, taking into account an expected attrition rate of 20%. However, across the 85 recruited participants, 19 were excluded during the screening session, either because they declined to participate (n = 4), or due to poor behavioral performance on our working memory task (n = 4), or due to contra-indications to rTMS (n = 11). Over the 66 remaining participants, 3 were excluded after the imaging visit due to noisy fMRI induced by movement, and 14 were excluded during the TMS visits due to scheduling reasons (n = 8) or because of pain due to the stimulation (n = 6). Finally, 2 other participants were excluded as they did not have complete data across the TMS visits, because of a lack of time; See Fig 1 for consort diagram). Twenty-nine young adults and eighteen older adults completed the full protocol (see Table 1 for baseline demographics). However, for some subjects, stimulation intensity and the position of the rTMS target had to be adjusted to increase tolerability (see the Results section). Participants had normal, or corrected-to-normal, vision and were native English speakers. Participants were compensated $20/hour for their efforts with a $100 completion bonus.

**Table 1:** Baseline demographics for young and older adults.

	Young Adults (n = 29)	Old Adults (n = 18)
**Age (Mean ± SD)**	22.9 ± 4.8	69.7 ± 4.8
**Number of Females**	17	13
**Number of Males**	12	5
**Number of education years (Mean ± SD)**	15.7 ± 2.59	17.3 ± 1.10

### Experimental protocol

**Fig 2:**
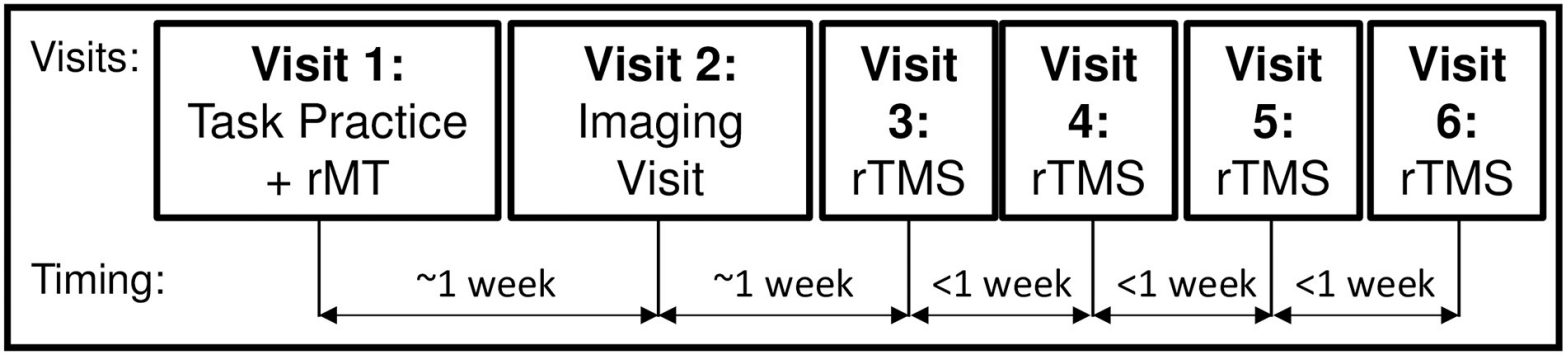
Illustration of the protocol describing the six visits and the relative time interval between each.

Participants were scheduled for 6 sessions (Fig 2). The first visit (Visit 1) consisted of consenting, exclusionary screening, resting motor threshold assessment and 6 blocks of practice with the behavioral task. Participants then returned for an MRI visit (Visit 2) and four more rTMS sessions (Visits 3–6). Visits 1, 2 and 3 were separated by approximately 1 week each, and on average Visits 3–6 were completed in 11 days.

#### Delayed-response alphabetization task (DRAT)

**Fig 3:**
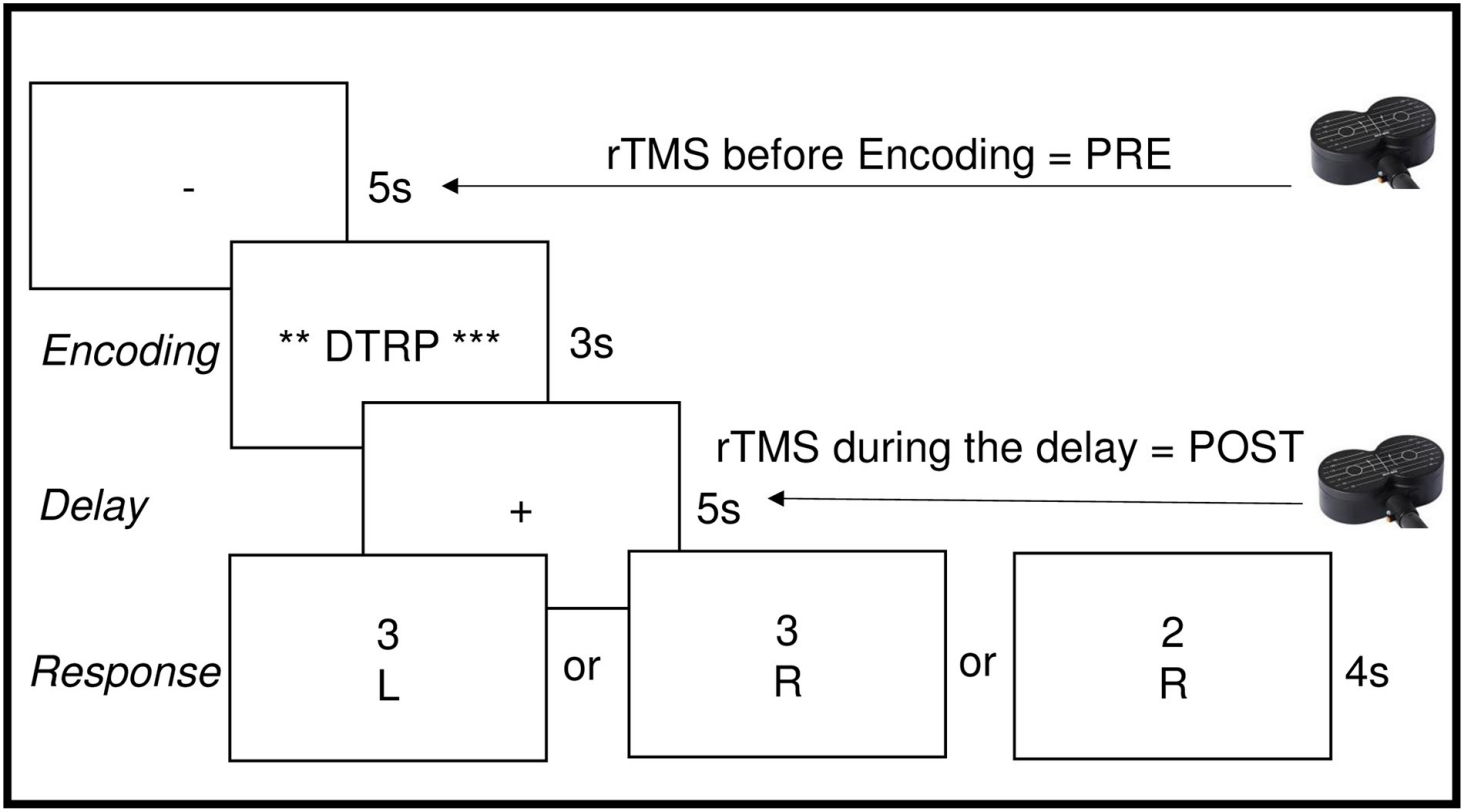
Schematic illustration of DRAT. One trial is shown with an array of 4 letters to encode, followed by a 5s delay period, during which participants had to maintain and reorganize the letters into alphabetical order. Examples of the 3 possible responses are shown at the bottom: “New”: the letter was not in the original array; “Valid”: the letter was in the array and the number represented the correct position in the alphabetical order; “Invalid”: the letter was in the array but the number did not match the correct serial position when alphabetized.

The delayed-response alphabetization task (DRAT: Fig 3) was designed to investigate working memory manipulation abilities. On each trial, an array containing 3 to 9 letters was presented on a screen for 3 seconds, followed by a 5-second delay period during which the participants were asked to keep this array in mind (maintenance) and to reorganize the letters into alphabetical order (manipulation). The set of letters included all English consonants, with vowels excluded to reduce spontaneous chunking. After the delay period, a letter with a number above it appeared on the screen for 4 seconds and participants were asked to report using one of three button options if (1) the probe letter was not in the original set, if (2) the letter was in the original set and the number matched the serial position of the letter once the sequence was alphabetized, or if (3) the letter was in the original set but the number did not match the serial position of the letter once alphabetized. These conditions are referred to as New, Valid and Invalid, and consisted of 20%, 40% and 40% of the trials respectively. For all three conditions, the probe was never from the first half of the alphabetized array, and in the Invalid condition, to exclude obvious differences between correct and incorrect position, the number above the letter was always within 1 step of the letter’s actual alphabetized position. During the first visit, the response phase was followed by a 5-second inter-trial interval during which the participants got feedback displayed as “Correct” or “Incorrect”, in green or red font respectively, depending on their performance during the previous trial; during the rTMS visits, no feedback was given during the delay period. For all visits (Visit 1, Visit 2, Visit 3–6), accuracy was displayed on the screen at the end of each block.

In the first visit, participants performed 6 blocks (150 trials) of the task using a 2-down-1 up staircase procedure. According to this procedure, the set size on each trial was increased by one item following each correct response and decreased by 2 following each incorrect response. Because this procedure was designed to establish task difficulty for the manipulation of information in the subsequent TMS sessions, the staircase procedure only took into account the performance on Valid and Invalid trials which were not expected to differ, while the New trials were considered catch trials and were excluded from the staircase adjustment procedure. To estimate set size difficulty levels for each individual, accuracy from the Valid and Invalid conditions were collapsed and fitted to a sigmoid function, using the Matlab (MathWorks, Inc., Massachusetts, USA) ‘sigm_fit’ function (https://www.mathworks.com/matlabcentral/fileexchange/42641-sigm_fit). The sigmoid function was calculated using the following formula:

f(x)=p1+(p2-p1)(1+10^((p3-x)*p4))

with f(x) the accuracy for each set size, x the set size, p1 the parameter determining how the fitted function is moved up/downwards on the f(x) axis; p2 the parameter limiting the sigmoid function on the f(x) axis; p3 the point on the x axis where the fitted curve changes direction; and p4 the steepness of the sigmoidal part of the curve.

To assure that the psychometric function was not strongly influenced by low numbers of trials at the easiest and hardest set sizes, 50% accuracy was used for the largest set sizes if less than 10 trials were tested. To achieve more stable curve fits, anchor points were added to both ends of the set size by accuracy plots, using 100% accuracy for set sizes of 1 and 2, and 50% accuracy for set sizes 10 and 11. The subsequent set sizes were defined relative to 82% correct, approximately the theoretical convergence point for this staircase [27], the intersection between the 82% accuracy threshold and the fitted sigmoidal function was then identified as the breakpoint for subsequent set size assignments. The two set sizes lower than the intersection values were defined as the Very Easy and Easy levels, while the two set sizes greater than this value were defined as Medium and Hard. All four levels were used in the subsequent MRI session, but the Very Easy level was not included in the TMS sessions to avoid ceiling effects and to increase the number of trials per condition in the study.

#### MRI acquisition

Participants were scanned on a 3-T gradient-echo scanner (General Electric 3.0 Tesla Sigma Excite HD short bore scanner), equipped with an 8-channel head coil. During this session, a structural MRI and a diffusion weighted imaging (DWI) scan were acquired, as well as functional acquisitions while participants performed 4 blocks of the DRAT. The anatomical MRI was acquired using a 3D T1-weighted echo-planar sequence (matrix = 256^2^, time repetition [TR] = 12 ms, time echo [TE] = 5 ms, field of view [FOV] = 24 cm, slices = 68, slice thickness = 1.9 mm, sections = 248). 3D T2-weighted, with fat saturation, echo planar sequence were also acquired (matrix = 256^2^, TR = 4000 ms, TE = 77.23 ms, FOV = 24 cm, slice thickness = 2 mm). Coplanar functional images were acquired using an inverse spiral sequence (64 × 64 matrix, TR = 2000 ms, TE = 31 ms, FOV = 240 mm, 37 slices, 3.8-mm slice thickness, 254 images). Finally, DWI data were collected using a single-shot echo-planar imaging sequence (TR = 1700 ms, slices = 50, thickness = 2.0 mm, FOV = 256 × 256 mm^2^, matrix size 128 × 128, voxel size = 2 mm^3^, b value = 1000 s/mm^2^, diffusion-sensitizing directions = 25, total images = 960, total scan time = 5 min).

Stimuli for the DRAT were back-projected onto a screen located at the foot of the MRI bed using an LCD projector. Subjects viewed the screen via a mirror system located in the head coil and the start of each run was electronically synchronized with the MRI acquisition computer. The DRAT was performed using the 4 titrated difficulty levels defined from Visit 1. Overall accuracy was presented on the screen at the end of each block of 30 trials. Behavioral responses were recorded with a 4-key fiber-optic response box (Resonance Technology, Inc.). Scanner noise was reduced with ear plugs, and head motion was minimized with foam pads. When necessary, vision was corrected using MRI-compatible lenses that matched the distance prescription used by the participant. The total scan time, including breaks and structural scans, was approximately 1 hour 40 minutes.

#### MRI processing

Functional images were skull stripped, reoriented and corrected for slice acquisition timing, motion, and linear trend using the FMRIB Software Library (FSL) image processing tools (https://fsl.fmrib.ox.ac.uk/fsl/fslwiki) including FLIRT and FEAT, available in a publicly available pipeline developed by the Duke Brain Imaging and Analysis Center (https://wiki.biac.duke.edu). Motion correction was performed using FSL’s MCFLIRT, and 6 motion parameters were then regressed out of each functional voxel using standard linear regression. Images were then temporally smoothed with a high-pass filter using a 190s cut off, and normalized to the Montreal Neurological Institute (MNI) stereotaxic space. Separate events were modeled for the array presentation (duration: 3s), delay period (duration: 5s), and response (duration: 4s), each with an onset at the beginning of the event. Incorrect and non-response trials were modeled identically, but separately. Parametric statistics were used to examine changes in the neural correlates of underlying WM manipulation, allowing activity to be modeled as a function of discrete changes associated with set size increases. These adaptive changes allowed us to model how responsive an individual would be to parametric variability in the set size across trials. At the first level, functional data were analyzed as individual runs. Second-level analyses combined data across runs for each subject using a fixed-effects model. Functional data were analyzed using a general linear model (GLM) in which trial events were convolved with a double-gamma hemodynamic response function. The GLM examined BOLD response during trials where the correct response was chosen in the behavioral task. The GLM included separate regressors modeling the duration of the array, the duration of the delay period, and the duration of the probe period. Additionally, weighted regressors were included during the delay period to model the parametric increase in difficulty with increased set size. These were orthogonalized with the delay period regressor. This processing allowed for the identification of individualized statistical maps that predicted the parametric increase in BOLD activity associated with increasing set size. The peak of activation within the left medial frontal gyrus in each participant was chosen as the rTMS target and entered into the neuronavigation system (BrainSight, Rogue Research, Canada).

#### Electric field modeling and TMS targeting

**Fig 4:**
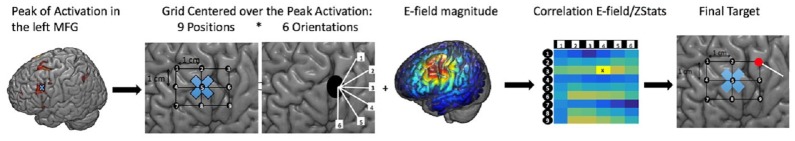
TMS targeting procedure illustrated. From left to right: Peak BOLD activation (1<z<3) on the left DLPFC associated with increasing set size; E-field grid with 3 × 3 positions and 6 coil orientations centered at the peak BOLD activation; representation of the E-field magnitude for one of the 54 options; correlation matrix between E-field magnitude for each of the 54 options and the activation z-values in which the highest correlation is for position 3 and orientation 4; optimized rTMS coil location (red dot) and orientation (white line).

TMS effects depend strongly on the spatial distribution of the electric field (E-field) induced in the brain, which determines what neural populations are affected [28]. Thus, simulation of the E-field induced by TMS in individual participants is increasingly recognized as an important step in spatial targeting of specific brain regions and forms a critical link between the externally applied TMS parameters and the neurophysiological response supporting cognitive operations. To determine the E-field induced by TMS in the brain of each participant, simulations using T1, T2, and DWI images and the finite element method in the SimNIBS software package [29] were conducted. The models featured five distinct tissue types: skin, skull, cerebrospinal fluid, grey matter, and white matter. The DWI information was used to generate anisotropic conductivities for white matter using the volume-normalized approach. The spacing between the coil and the scalp was assumed to be 4 mm (the default for SimNIBS). To select the position of the figure-8 coil for rTMS across 3 locations and 3 angular parameters, E-field models were used to determine the maximum overlap between the E-field strength and activations from the fMRI task (Fig 4). The optimization focused on the E-field strength since it appears to be the key determinant of neural recruitment by TMS [30]. The E-field was simulated at 54 coil targets (9 positions and 6 orientations per position) with a model of the figure-8 coil used in the study (B65, MagVenture, Denmark). The 9 positions were generated by placing a 3 × 3 grid with 1 cm^2^ spacing above the peak fMRI activation. For each position, six different coil orientations were simulated corresponding to 30° rotation increments in a 180° semicircle. Due to the symmetry of the E-field, the 180° semicircle was sufficient to encompass all orientations in the full 360° circle. The E-field magnitude distributions, constrained to a magnitude higher than 0.4 V/m, for each of the 54 coil targets were correlated with the positive z-values of the fMRI activation. The coil position and orientation with the highest correlation was selected as the primary rTMS target, while the second and third highest correlations were noted as backup targets in the event that the primary target could not be used, for instance due to tolerability issues.

#### TMS procedures

TMS was performed with an active/placebo figure-8 coil (A/P Cool-B65) and a MagPro X100 stimulator with MagOption (MagVenture, Denmark), while the coil position was continually monitored through a stereotaxic neuronavigation system (Brainsight, Rogue Research, Canada) and was maintained at a high level of precision throughout the session with real-time robotic guidance using the Smart Move Robot (Advanced Neuro Technology, Netherlands). The TMS device was configured for biphasic pulses, in standard pulse width mode with the direction of the TMS pulses such that the initial phase of the E-field pointed from anterior to posterior direction.

To determine the motor threshold, electrodes (Neuroline 720, Ambu) were placed on the FDI in a belly—tendon montage and motor evoked potentials (MEPs) were recorded by an electromyogram (Power Lab and LabChart). On the first visit, motor hot spot was defined as the position over the left motor cortex that elicited the greatest MEP in the right first dorsal interosseous (FDI). Resting motor threshold (rMT) was then defined as the TMS pulse intensity producing on average an MEP of 50μV peak-to-peak amplitude, using a maximum likelihood estimator (TMS Motor Threshold Assessment Tool, MTAT 2.0, http://www.clinicalresearcher.org/software.htm) [31].

From visit 3 to 6, participants performed the DRAT while active or sham rTMS was delivered to the identified left DLPFC target. Twenty-five pulses of 5Hz rTMS were delivered at 100% of rMT on each trial, either immediately before encoding (Pre) or post-encoding, during the delay period of the DRAT (Post), see Fig 3. Sham stimulation was applied using the same coil (A/P Cool-B65) in placebo mode, which produced similar clicking sounds and somatosensory sensation (via electrical stimulation with scalp electrodes) as in the active mode, but without a significant magnetic field reaching the brain. This type of sham stimulation allows participants to stay blinded during the course of the experiment. To test the efficacy of the blinding without revealing to the participants that there was an active and sham condition, participants were asked at the end of each visit: “Do you think that rTMS affected your performance”. None of the subjects reported a block differences, suggesting that they did not notice any differences between the two types of stimulation.

On each visit, subjects performed the DRAT with the titrated 3 difficulty levels (Easy, Medium, and Hard), with accuracy feedback given at the end of each block. Ten blocks of the DRAT task were performed: a first block without stimulation (NoStim1), four blocks of active or sham stimulation, one block without stimulation (NoStim2), and four more blocks with the sham or active stimulation. The order of stimulation type (Active or Sham) was presented according to an ABBA schedule that repeated twice (one cycle during the first two sessions and one cycle during sessions three and four), while the stimulation timing (Pre or Post) alternated from block-to-block and flipped order between the 2nd and 3rd session. The starting order for stimulation type and stimulation timing was counterbalanced across participants. Random allocation, enrollment and assignment was made through a Matlab script that was administered by the Clinical Research Specialists.

### Tolerability

In addition to the six subjects who stopped participation in the study because of pain, deviations from the planned stimulation protocol occurred for 20 other subjects who completed the full study. For 16, the intensity of rTMS was reduced to alleviate discomfort (with relative intensities ranging from 68 to 99% of the initial intensity). In addition, for 10 subjects (six of whom also required intensity reduction), the placement of the coil was adjusted by selecting the backup e-field target (n = 7), the second backup E-field target (n = 1) or the target defined by fMRI activations rather than the target defined by the E-field (n = 2). Because performance of these participants were not outliers from group means, when comparing the overall accuracy in the active and the sham condition they were kept in the analysis.

### Data preprocessing

The main analyses of interest in this study concerned the influence of rTMS on WM manipulation abilities. In order to infer these relationships, several analytical steps were taken. First, behavioral performance was defined by focusing on trials and conditions of interest. As such, data from the first three trials of each block were removed, as participants were asked to provide feedback on the somatosensory effects of rTMS and thus did not focus on the task. ‘New’ trials did not involve WM manipulation and these trials were not included in subsequent analyses. Furthermore, given that the difficulty levels were individually titrated, difficulty level was normalized according to the starting set size for each participant. One of the older adults was excluded from the analysis, as his overall accuracy was an outlier from accuracy group means. The final analysis on accuracy, were thus performed on 46 subjects (29 young and 17 older adults). Reaction times were not emphasized in this experiment, as they are associated more with the decision process rather than the WM manipulation which serves as the focus of the present study.

## Results

### Cumulative effect of rTMS

In the current design, active and sham stimulation were performed on the same day. To ensure that no cumulative “carryover” effect could have contaminated the effect of one type of stimulation over the other, we first analysed the block of trials performed either before the stimulation (NoStim1), or after each type of stimulation (NoStim2_AfterActive, and NoStim2_AfterSham). One way ANOVA, performed on the average accuracy across these three blocks, did not reveal any differences between blocks performed before stimulation (NoStim1 = 67.65 ± 1.44%), blocks performed after active rTMS (NoStim2_AfterActive: 67.53 ± 1.7), or blocks performed after sham rTMS (NoStim2_AfterSham: 69.11 ± 1.7; F(2,90)<1). This result suggests that no carryover rTMS effect persisted and thus that subsequent rTMS effects were not contaminated by the former type of stimulation.

### Absolute vs. Relative set sizes

In this study, the number of memory items were individually titrated based on participant’s performance during the first visit. As such, the set sizes for each participant can be considered in terms of the absolute number of letters to be remembered, or in terms of the relative number of items based on that person’s titrated set sizes. Preliminary analyses were performed to determine if differences existed between young and older adults on the absolute set sizes, allowing insight into whether the two age groups differed in their baseline task performance. An independent samples t-test was performed on the Starting Set Size, i.e., the number of letters in the array for the Easy set size. The results did not reveal any differences between the groups (Older adults: 4.76 ± 0.56 vs. Young adults: 5.00 ± 0.76 (t(44) = -1.11; p = .27). A factorial ANOVA was also performed on accuracy with Starting Set Size and Group as independent variables. This revealed a significant main effect of Starting Set Size (F(2,40) = 9.48, p < .01), but no significant differences between Groups (F(1,40) <1) or interaction between Groups and Starting Set Size (F(2,40) = 1.05; p = .36) were found. As such, it can be inferred that the two groups performed roughly equivalently and consequently, absolute Set Sizes were not included in subsequent analysis.

### rTMS effects on the DRAT

To assess rTMS effects on accuracy during the DRAT, an ANCOVA was run for each trial type (Valid and Invalid). Indeed, it can be argued that the cognitive processes involved in these trials differ because Invalid trials require additional cognitive control to reject the incorrect pairing of the letter with the invalid probe number. Thus, for each trial type, average accuracy from each participant was entered in an ANCOVA with Group (Old and Young adults) as the between-subject factor, and the following within-participant factors: Visit (Visit 3, Visit 4, Visit 5 and Visit 6), Difficulty (Easy, Medium, and Hard), Stimulation Timing (Pre and Post) and Stimulation Type (Active and Sham). Normality, tested using Kolmogorov-Smirnov test, was respected. Neither of these ANCOVAs revealed main effects of Group or an interaction between Group and Stimulation Type. As such, to reduce the number of factors, data from both groups were collapsed, and an ANOVA was then used with the following factors: Visit (Visit 3, Visit 4, Visit 5 and Visit 6), Difficulty (Easy, Medium, and Hard), Stimulation Timing (Pre and Post) and Stimulation Type (Active and Sham). The following section describes the results from the ANOVA performed on the Invalid trials, and on the Valid trials.

#### rTMS effects on the DRAT task for Invalid trials

When considering the more demanding Invalid trials, a significant main effect of *Visit* (F(3, 132) = 10.71, p < .001, η^2^ = 0.19) was found with better accuracy on the two last visits compared to the two first ones (all ps < .01), suggesting a learning effect across time. A significant main effect of *Difficulty* (F(2,88) = 183.26, p < .001, η^2^ = 0.81) was also present, with better performance on the Easy, compared to the Medium and Hard difficulty levels (p < .01 for all pairwise comparisons). The main effects of *Stimulation Timin*g and *Stimulation Type* (all Fs(1,44)<1), however, were not significant. (See Table 2 for the average accuracy and standard error for all the significant main effects).

**Table 2:** Summary of mean accuracy and standard error for the significant main effects.

	Mean accuracy and standard error (in percentage) for each factor levels for the Invalid trials			
Visits:	Visit 3: 59.44 (7.24)	Visit 4: 61.31 (7.18)	Visit 5: 65.36 (7.02)	Visit 6: 65.98 (6.99)
Difficulty	Easy: 79.49 (5.95)	Medium: 60.78(7.2)	Hard: 48.74 (7.37)	__

**Fig 5:**
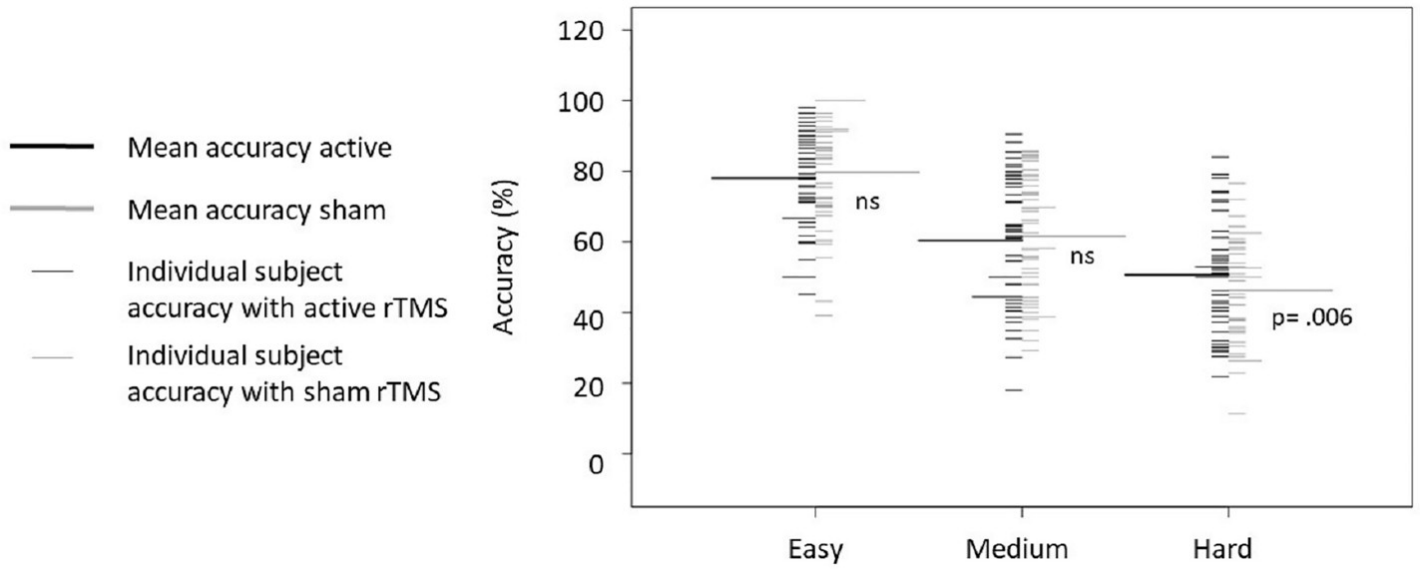
Mean accuracy in the Invalid trials. Accuracy for active (black) and sham (grey) rTMS are displayed for each difficulty level. Small lines represent individual data. The longer lines represent the average for each condition.

As expected, a significant interaction between *Difficulty* and *Stimulation Type* was found (F(2, 88) = 8.49, p < .001, η^2^ = 0.16). The decomposition of this interaction with Bonferroni correction revealed a significant difference in the Hard condition, with active rTMS producing increased accuracy (50.43 ± 7.37%) compared to sham rTMS (46.49 ± 7.35%; F(1,44) = 17.15, p = .006). However, no significant differences were found between active (78.04 ± 6.11%) and sham stimulation (80.31 ± 5.86%) in Easy (F(1,44) = 2, p = 1), or Medium difficulty conditions (60.58 ± 7.21% vs. 61.82 ± 7.16%, F(1,44) = 1.05, p = 1; Fig 5 and Table 3).

**Table 3:** Summary of mean accuracy and standard error. Accuracy for Valid and Invalid trials for each active and sham stimulation at each difficulty level (Easy, Medium and Hard) are presented. P-values represent the interaction between Difficulty and Stimulation Type derived from the ANOVA performed for the Invalid condition. Interaction for the Valid condition was not significant and is not shown here.

Difficulty:	Easy			Medium			Hard		
Stimulation Type:	Active	Sham	p-values	Active	Sham	p-values	Active	Sham	p-values
Valid Trials:	86.26 (5.08)	84.09 (5.39)	NA	70.29 (6.74)	72.30 (6.60)	NA	59.32 (7.24)	58.53 (7.27)	NA
Invalid Trials:	78.04 (6.11)	80.31 (5.86)	NS	60.58 (7.21)	61.82 (7.16)	NS	50.43 (7.37)	46.49 (7.35)	.***006***

#### TMS effects on the DRAT task for valid trials

Analyses of the valid trials revealed main effects of *Visit* (F(3,132) = 11.14, p < .001, η^2^ = 0.20) and *Difficulty* (F(2,88) = 118.8, p < .001, η^2^ = 0.73). Post-hoc analyses performed with Bonferroni correction revealed that accuracy in the two last visits were significantly higher than accuracy during the two first ones (all ps < .01). In addition, there were significant reductions in accuracy with larger set sizes for all pairwise comparisons. A significant main effect of *Stimulation Timing* was also found (F(1,44) = 4.45, p = .041, η^2^ = 0.09), with better accuracy when stimulation was applied before encoding, relative to TMS applied during the delay. The effect of *Stimulation Type* (F(1,43)<1) was not significant. No significant interactions were present. (See Table 4 for the average accuracy and standard error for all significant main effects).

**Table 4:** Summary of mean accuracy and standard error for the significant main effects.

	Mean accuracy and standard error (in percentage) for each factor levels for the Valid Trials			
Visit:	Visit 3: 67.91 (6.88)	Visit 4: 70.56 (6.72)	Visit 5: 74.7 (6.41)	Visit 6: 74.82 (6.40)
Difficulty:	Easy: 85.65 (5.17)	Medium: 71.18 (6.68)	Hard: 59.02 (7.25)	_________________
Stimulation Timing:	Pre: 72.5 (6.58)	Post: 71.12 (6.68)	_______________	________________

#### Summary of rTMS effects and ancillary results

**Fig 6:**
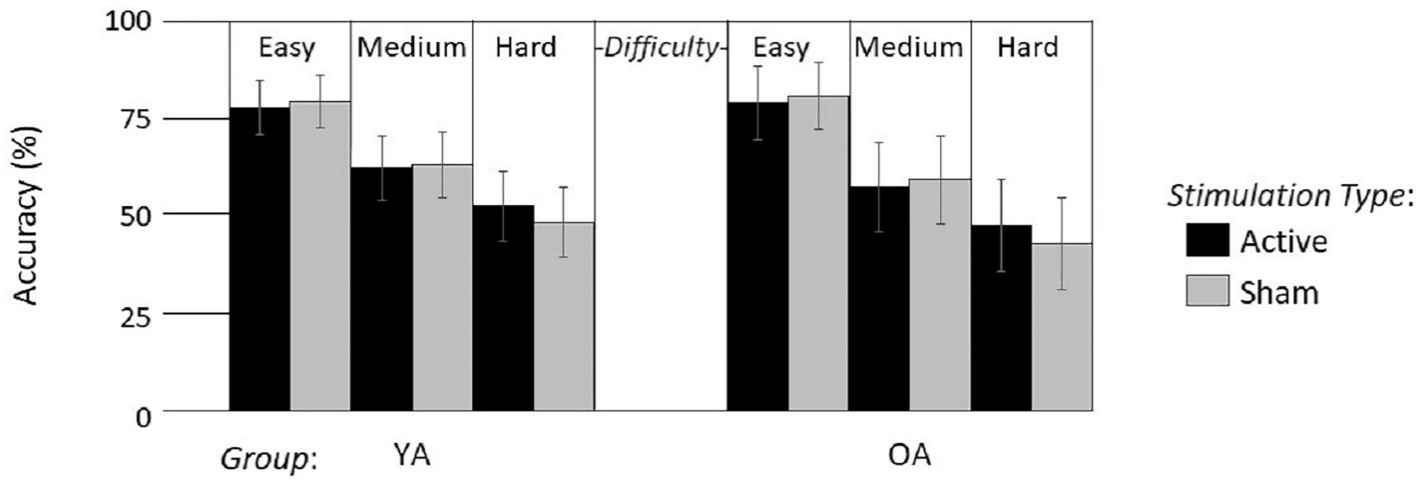
Mean accuracy in the Invalid trials for active (dark grey) and sham (light grey) rTMS and for each difficulty level, for young (YA) and older adults (OA). Error bars represent the standard error.

Overall, these results revealed that rTMS delivered to the DLPFC, identified with fMRI and e-field modeling, enhanced WM manipulation in the combined young and older adult group. It is notable that this effect was found only in the most difficult condition, that is, in the hardest difficulty level of Invalid trials. Nonetheless, when investigating this effect in both groups separately, the one-way interaction between Difficulty and Stimulation Type, in the Invalid trials, was found to be significant for both the young (F(2, 54) = 4.11, p = .022, η^2^ = 0.13) and the older cohort (F(2, 32) = 4.79, p = .015, η^2^ = 0.23; Fig 6). As such, while it is surprising that no such effects were seen in the less difficult conditions, the presence of behavioral facilitation in the hardest condition for both groups is consistent with a priori hypotheses, demonstrates generalization across populations, and warrants further study.

As an ancillary finding, a main effect of *Stimulation Timing* was found only during the valid trials, when the task was easier to perform. As this effect did not interact with *Stimulation Type*, it suggests that the somatosensory sensations induced by rTMS could either improve performance when applied before the encoding, or disrupt performance when stimulation was applied during the delay. This result could be a side effect of somatosensation, which has been formerly shown to correlate with error rates during a delay match to sample task [32]. The current design, however, did not allow strong conclusions about the direction of this effect. Interestingly, this effect was not observed in the Invalid condition, the most difficult task condition, which suggests that nonspecific TMS effects can disappear when the task is harder.

### E-field targeting

**Fig 7:**
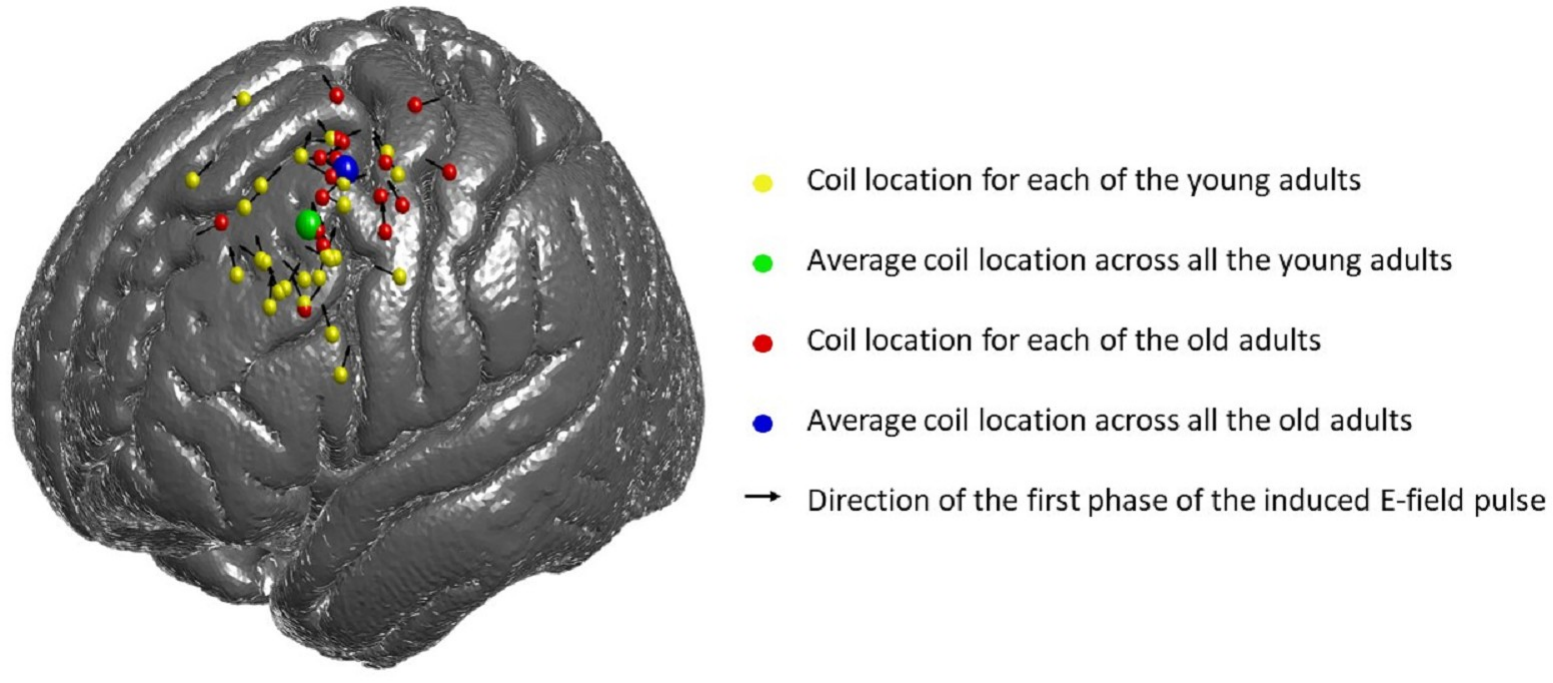
TMS coil position (spheres) and orientation (arrows) for each subject. The average coil location is displayed in yellow for the young, and in red for the older group. The spheres represent the coil location and the arrows correspond to the direction of the first phase of the induced E-field pulse (some of the arrowheads are not visible because of the 3D view). The green and the blue sphere represent the average coil location across all subjects, for the young and the old group, respectively.

As described in the methods section, this study uses a novel approach for TMS targeting based on optimized coil location and orientation that induce the strongest E-field exposure over regions of the brain, within the PFC, that show the highest parametric fMRI activation during the WM task. The logic of this approach was that by selecting the optimization solution producing the highest correlation between E-field magnitude and positive fMRI z-score, this selection would take into account each participant’s individual physiology to stimulate the most relevant brain regions, presumably leading to the strongest TMS effect. It is important to consider that this novel, highly individualized approach for TMS targeting results in a wide range of placements and orientations, as shown in Fig 7. While this variability is consistent across younger (yellow) and older adults (red), targeting variability may contribute to the moderate effects obtained in this study.

## Discussion

The goals of this study were, first, to test if online high frequency rTMS to DLPFC could enhance WM manipulation and if this differed as a function to task difficulty. The second goal was to investigate the effect of age on those rTMS effects. The results yielded two main findings. First, active 5Hz rTMS delivered to individualized left DLPFC targets, identified with fMRI and e-field modeling, significantly enhanced WM manipulation abilities over the course of the intervention, but only in the most difficult condition of the task, namely the hardest difficulty level of the Invalid trials. Second, active versus sham rTMS produced similar effects in young and older adults, suggesting that rTMS enhancement was not modulated by age. Each of these findings are discussed in separate sections below.

### rTMS of WM manipulation and difficulty effects

Although rTMS was initially used to disrupt performance by generating neural noise during the cognitive processes [33], studies indicated that rTMS could also lead to performance enhancement [34]. This opened the possibility that rTMS could be used to enhance WM in cases of cognitive impairment, which is supported by remediation of WM in sleep deprivation [16, 17]. To our knowledge, only a few studies have used online rTMS to enhance WM in healthy participants [11–13]. While these studies showed promising results with shortened reaction times associated with active rTMS applied over the parietal cortex, these studies have only focused on the maintenance component of WM. In the current study, we were interested in the manipulation component of WM because of the critical role it plays in executive cognitive functions and because of the notable declines in this ability associated with healthy and disordered aging. Here the DLPFC was chosen as the stimulation target since it is widely accepted that DLPFC mediates WM manipulation. While consistent evidence implicates parietal areas in the actual storage of information [35], the DLPFC appears to be more involved in the manipulation of information [36], particularly when cognitive load increases [37]. The present finding that rTMS can modulate performance in the DRAT, therefore, supports the assertion that DLPFC plays a role in the manipulation of information within WM.

In this study, performance enhancements associated with active compare to sham rTMS were found only in the most difficult task condition: the hardest difficulty level in the Invalid trials. Recent studies have shown the rTMS effects are state-dependent, i.e. vary according to the activation state of the stimulated neuronal population [38]. For example, using psychophysical adaptation paradigms to modulate the initial activation of neural populations, it has been shown that TMS facilitates the less active neural populations relative to the more active neural populations [39, 40]. Although these studies were based on sensory tasks, it seems possible that brain state could also have an impact during more demanding cognitive tasks. These results are in line with a priori hypotheses that rTMS enhancement would be greatest in the most challenging conditions, as reported in previous studies [11, 16–18], and further confirms this state-dependency assumption. Nonetheless it is surprising that effects were seen only in the Invalid trials. During these trials, participants were asked to reject remembered items while, during Valid trials, they were to accept them. Performing Invalid trials is more cognitively demanding as it requires additional executive control required to reject the multiple possible incorrect parings [41,42], which may tie into state-dependency and contribute to the pattern of observed effects. In this study, brain activations associated with the difference between Valid and Invalid trials were not investigated. Indeed, the delay phase of the task was used to investigate fMRI activations for the rTMS targeting, however differences between Valid and Invalid trials would arise only during the response phase. Future studies may wish to investigate differences in brain activations associated with these two types of trials, which could allow a better understanding of the differential rTMS effect across these two conditions which might enable the production of a larger effect size on performance enhancement.

### rTMS and aging

The second goal of this study was to investigate the modulatory effect of aging on rTMS effects. Surprisingly, even though it is widely accepted that healthy aging is associated with decline in cognitive performance, very few studies have investigated the ability of rTMS to enhance cognitive abilities in this population, and no studies have compared this effect of rTMS between young and older adults. Healthy aging has been shown to be associated with a variety of brain changes such as decreases in GABAergic neurotransmission [43] and decreases in neuroplasticity [44], as well as by the action of compensatory mechanisms as reflected by a reduction in hemispheric lateralization and the recruitment of additional cortical resources [45]. While the exact mechanisms underlying rTMS effects remain largely unknown, they have been linked to these factors. Consequently, one could assume that the aging brain could react differently to rTMS compared to younger brains. This study did not reveal any differences between young and older adults in terms of behavioral performance, nor in terms of rTMS effects. These results therefore suggest that rTMS could be used to enhance cognitive abilities in healthy aging. However, performance enhancement observed in this study was modest, and more importantly stimulation induced discomfort for some of the subjects. Therefore, more investigation is needed before using rTMS in larger population, for example by targeting other brain areas that are less sensitive, such as posterior brain regions.

### Strengths and weaknesses

This study reports promising, but modest rTMS effects that are not present across all conditions. As such, it is important to consider the strengths and weaknesses of this design and modifications that may possibly increase effect sizes in future studies. In particular, two main points should be considered; participants’ compliance (and deviations) from the protocol, as well as the highly individualized nature of this protocol.

With regard to compliance, in the current protocol a number of slight deviations from the planned protocol in terms of intensity of stimulation and selected target occurred. These adjustments were made because of rTMS-induced discomfort, but were also considered in the analyses to assure that large deviations were not driving the primary findings. Moreover, a relatively large number of individuals did withdraw from the study. While the majority of these were due to scheduling conflicts, it does speak to the relative opportunity costs of implementing such a 6-session protocol. This suggests that this protocol involving brain imaging, brain stimulation over the prefrontal cortex, and a total of six visits is taxing for participants, and possibly might be adjusted to allow for easier recruitment, and less deviations.

Although this protocol was challenging to apply, it does possess several strengths. In particular, the design was extensively tailored on a participant-by-participant basis. In terms of the behavioral task, difficulty levels were individually titrated through a staircase procedure. This potentially reduced state-dependency that would be caused by fixed difficulty levels that do not match individual capabilities. In terms of stimulation, rTMS targets were defined using individualized fMRI activations, and computational modeling was used to select the TMS coil location and orientation inducing the strongest electric field exposure of the target. Additionally, stereotactic neuronavigation and robotic real-time guidance were used to position the TMS coil. While individualized fMRI-guided TMS has been shown to induce larger effect sizes relative to anatomical or group targets [46], very little is known about the impact of fMRI-targeting combined with electric field modeling; this approach is relatively new and involves additional assumptions that may be modulating effects. The targeting approach in this study used the strongest correlation between positive fMRI activation and electric field magnitude to define the optimal coil location and orientation among 54 different options. As displayed in Fig 7, this approach led to considerable variability in target location, and therefore future studies may wish to expand upon this approach or test other targeting approaches to evaluate efficacy.

Finally, despite the large number of individualized parameters in this study, both the stimulation intensity (100% resting motor threshold) and the frequency of stimulation (5Hz) were fixed. These parameters were selected based on previous results showing that rTMS was effective to enhance working memory maintenance [11, 13]. rTMS has been shown to entrain endogenous task-related oscillatory dynamics [19], and given the role of theta oscillations in memory processes [21], 5Hz rTMS was believed to be the most appropriate frequency of stimulation. However, futures studies may wish to vary these parameters. For example, by using closed-loop information from simultaneously-recorded EEG it may be possible to individualize the stimulation frequency and potentially achieve larger effect sizes. Finally, it has been proposed that performing tasks while receiving rTMS to activated brain areas, at a frequency matching the ongoing natural brain oscillations associated with the task would result in stronger rTMS effects through Hebbian-like plasticity [47, 48]. However, the design used in this study did not directly test this assumption.

## Conclusion

The current study showed that 5Hz rTMS applied over the left dorsolateral prefrontal cortex can enhance WM manipulation abilities, but only in the most difficult condition, with no differences between young and older adults. This result points towards further investigation of rTMS for memory enhancement, focusing on high difficulty conditions as those most likely to exhibit benefits.

## Supporting information

S1 File Consort checklist. (DOC)

S2 File Research summary. This document provides information about the study, the assumptions, and the inclusion and exclusion criteria.
(DOCX)

S3 File TMS adult safety screening. (DOC)

S4 File Detailed inclusion and exclusion criteria. (DOCX)

S5 File Adverse effect monitoring plan. (PPTX)
