# Supplementary material for: Online repetitive transcranial magnetic stimulation during working memory in younger and older adults: A randomized within-subject comparison
Source: PLoS One. 2019 Mar 22;14(3):e0213707. doi: 10.1371/journal.pone.0213707 (PMC6430375; doi:10.1371/journal.pone.0213707)
Supplement: S2 File — This document provides information about the study, the assumptions, and the inclusion and exclusion criteria. (DOCX) [file pone.0213707.s002.docx]

Research Summary

**1. Protocol Title:** Using fMRI-guided rTMS to increase executive control processing in older adults

*Note*: This protocol covers the first study (and Aim 1) of the NIA R01 grant supporting this research. A second protocol will be submitted covering the second and third studies and Aims.

**2. Purpose of the Study:**

Behavioral research on aging has mapped contrasting patterns of decline and stability in cognition across the adult lifespan. Both cross-sectional and longitudinal studies find robust declines in working memory (WM), which describes the storage and manipulation of temporary representations of information that was just experienced or just retrieved from long-term memory. The current proposal seeks to use a functionally-guided brain-stimulation (repetitive transcranial magnetic stimulation or rTMS) paradigm to modulate the working memory functions of dorsal lateral prefrontal cortex (DLPFC) or occipital parietal cortex (OPC). The research team carries the expectation that, since older adults rely more heavily on prefrontal function than young adults, older adults will benefit more from DLPFC stimulation than the young. Differential rTMS modulation of WM performance between young and older groups will provide causal evidence for compensatory processing, and help fill out our understanding of the neural basis of sustained, successful performance in aging. While we have previously demonstrated WM effects from stimulating the lateral occipital cortex (LOC), a specific region within the OPC, a finding of relative enhancement of executive processing with DLPFC stimulation- an enhancement that is long lasting and that transfers to executive processing beyond WM- is a key to development of noninvasive brain stimulation treatment of cognitive deficits, as this more frontal brain region is closely associated with neuroplastic abilities like executive processing. We have 3 key objectives:

**Primary Objective:** To evaluate the ability of rTMS to enhance working memory performance in both younger and older adults.

**Primary Endpoint:** We expect performance enhancements between active and sham rTMS to DLPFC or OPC to increase as the task becomes more difficult, as executive control processes are centered in this region.

**Secondary Objective:** To evaluate the selectivity of DLPFC in the enhancement of working memory performance. To accomplish this objective we will stimulate an alternative region (OPC), in both young and older groups,

**Secondary Endpoint:** We expect performance enhancements in the WM task between active and sham rTMS to remain constant in size across difficulty levels, as executive control processes are not centered in this region.

**Tertiary Objective:** To evaluate the selective benefit for older adults of rTMS stimulation to DLPFC or OPC during a working memory task.

**Tertiary Endpoint:** With DLPFC or OPC stimulation, the older group will show greater performance enhancements relative to sham than the young group, evidence for prefrontal compensatory processing in aging.

**3. Background & Significance:**

Cognitive decline and dementia have become important public health issues in our time as medical science has increased lifespan and our society becomes progressively older. While medical research has rightly focused on more serious dementias such as Alzheimer's disease, all individuals experience some measure of cognitive decline as they age (Harada et al., 2013). A great deal of the cognitive decline due to aging can be explained by decline in WM (Braver & West, 2010; Moscovitch and Winocur, 1992; Salthouse, 1994), a cognitive mechanism that enables humans to maintain and manipulate a limited amount of information for a brief period of time (see Hillary et al., 2006 for a review). WM is a critical cognitive function, and as such, WM plays a role in almost all aspects of cognition (e.g., fluid reasoning: Engle et al., 1999; language: Gathercole & Baddeley, 2014; multitasking ability: Gazzaley et al., 2012). WM is central to how information is being brought in and out of long term storage, within an ongoing spatio-temporal context, according to an individual's strategies (Courtney, 2004).

Deficits in WM have a great impact on cognitive function, and a large amount of the variability in cognitive deficits in the elderly has been correlated with WM impairment (Rozas et al., 2008; Zimprich and Kurtz, 2013). While earlier accounts focused on the age-related decline in working memory span, or the maintenance of information (Hambrick and Engle, 2002), more recently a number of studies have related age-related decline in a number of functions to a more general deficit in the ability to manipulate information. For example, the WM tasks that do show the most robust age differences are tasks that require the integration of short-term storage with executive control processes (e.g., manipulation of stored content as in backward digit span, or coordination with other complex cognitive computations such as mental arithmetic in operation span; Bopp & Verhaeghen, 2005; Myerson, Emery, White, & Hale, 2003). Consequently, current research on aging in WM has tended to focus on the executive control components WM tasks as a primary source of age differences. Recent work has been influential in pinpointing the cortical regions mediating these higher cognitive functions in both younger and older adults, including frontal pole and DLPFC (Duncan et al., 2005). Given the importance of WM to other aspects of cognitive function, studying the impact of aging on WM circuitry could lead to an enhanced understanding of overall cognitive deficits and a potential means to mitigate them.

The use of noninvasive brain stimulation, and in particular repetitive transcranial magnetic stimulation (rTMS), is a particularly effective means of carrying out such studies. This is due to the fact that the interactions of rTMS and the brain generate causal information, while other means such as brain imaging are merely correlational in nature. Further, these interactions can be beneficial, causing for example enhancements in cognitive performance or reductions in cognitive deficits, potentially leading to therapeutic uses. Over the past decade, it has become clear that rTMS can enhance cognitive performance, and we have specifically used it to enhance WM, developing an fMRI-guided rTMS paradigm that has replicated WM enhancement effects (Luber et al., 2007; Luber et al., 2008), including in older adults.

Previously, we have used an fMRI-guided rTMS paradigm to investigate enhancement effects on maintenance components of WM. Specifically, 5 Hz rTMS (approximately 7s, 35 pulses) applied immediately before each trial to the left LOC, a region involved with maintenance of items in WM. The choice of stimulus frequency is critical in rTMS studies; low frequency rTMS (e.g., 1 Hz) has typically been associated with a reduction in the activity in the target site (Luber, 2014), while high-frequency rTMS (> 5 Hz) have been associated with local excitation and improved cell-to-cell communication via long-term potentiation (Ziemann, 2004). Critically, our application of 5 Hz rTMS in these subjects improved WM performance in both young and older adults (Luber et al., 2007). While these effects are promising, most research attributes the aging effect on WM to be the breakdown of executive processes involved in manipulation components of WM, mediated by networks centered on DLPFC. In a key experiment (Luber et al., 2008), we generated temporary WM deficits in healthy young adults by sleep deprivation, and used our rTMS paradigm to remediate those deficits- an effect which lasted at least a day beyond the last rTMS application, and that these effects were mirrored in cortical changes fund using fMRI. The present proposal seeks to continue working with older adults, specifically targeting improvement of executive processes associated with WM, with the intent of demonstrating that our method of remediating WM can create persistent improvements in WM in older adults, and that by stimulating DLPFC, these improvements will transfer more generally to other cognitive tasks. These results will lay the foundation for possible therapies for cognitive decline in aging using functionally-targeted rTMS combined with behavioral training.

**4. Design & Procedures:**

This blinded, sham-controlled trial will feature a mixed model 2 x 3 x 2 x 2 cross-over design, with rTMS Type (active, sham), Difficulty Level defined by accuracy (fit with a sigma curve to define easy, medium, hard levels), Stimulation Location (DLPFC, OPC), and Group (Young, Older adults) as factors. As such, this design reflects the fact that all subjects receive both active and sham rTMS applied to up to two different scalp locations during a WM task at up to three levels of difficulty. Study procedures are listed in **Table 1**. Thirty healthy young adults (age range: 18-35 years) and thirty healthy older adults (age range: 55-80 years) will participate. In the initial session, which will take about 2 hours, participants will be consented and screened for the study, determine TMS motor threshold, and then learn and practice the WM task. The task will proceed through a staircase of difficulty in order to calibrate Difficulty Level to each participant. The next visit will occur within approximately 1 week (**Figure 1**) and involve a structural MRI and fMRI recorded from young and elderly groups while they perform the WM task. Cortical locations within OPC and DLPFC to be used as targets for rTMS will be chosen from an analysis of subject-specific fMRI activations from the WM task completed in this MRI Session. This session, which will last approximately 2 hours, will be followed by up to four sessions of rTMS, each lasting about 2 hours. These four TMS sessions will begin ~1 week after the MRI Session, to allow for time to process the imaging data, and within a 3 month period (Mondays thru Fridays), at the same time each day (+/- 2-3 hours for scheduling flexibility). Four rTMS sessions (involving active or sham rTMS and OPC or DLPFC targets) will use coil locations and rTMS device intensities based on realistic head modeling. Participants will report number of hours slept the previous night at each visit.

**Table 1. TIMELINE OF STUDY PROCEDURES FOR MAJOR STUDY:**

| **Assessment** | **Screening Session** | **MRI Session** | **rTMS Session 1** | **rTMS Sessions 2-4** |
| --- | --- | --- | --- | --- |
| 1. Subject consent & screening | X |  |  |  |
| 2. Practice with WM task | X |  |  |  |
| 3. Structural MRI |  | X |  |  |
| 4. fMRI with WM task |  | X |  |  |
| 5. Motor Threshold | X |  |  |  |
| 6. WM task + rTMS |  |  | X | X |
| 7. Side Effect Checklist |  |  | X | X |
| 8. Visual Analog Scale |  |  | X | X |

**
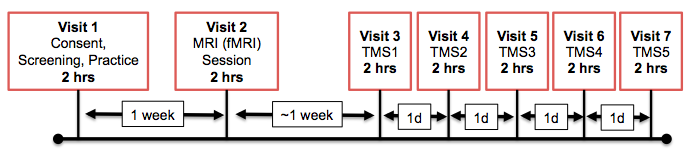
**

~

**Figure 1.** Timeline and timing of study procedures for the major study.

The above design and procedures overview will accomplish the goals of Aim 1 of the NIA R01 grant. In order to ensure the success of this design, we will precede the major experiment with a smaller pilot experiment to establish the best TMS timing parameters during our novel WM task. Up to 30 subjects will be recruited- 15 young adults (18-35 yrs), and 15 older adults (60-80 yrs; with the same inclusions and exclusions as the main experiment). Subjects will first undergo the screening and practice session (2hrs). One week later subjects will participate in two TMS sessions, one using active, and the other sham TMS, applied to DLPFC or OPC. This piloting stage of the project will allow us to proceed to the major experiment with confidence in our TMS timing parameters, and ensure that the protocol is optimized. Thus, the piloting stage differs from the major experiment in that a) there will be no MRI session, nor any other MR-related procedures, for this initial group of pilot subjects, and b) participants will participate in 2 instead of four TMS sessions.

**Table 2. TIMELINE OF STUDY PROCEDURES FOR PILOT STUDY:**

| **Assessment** | **Screening Session** | **rTMS Session 1** | **rTMS**  **Session 2** |
| --- | --- | --- | --- |
| 1. Subject consent & screening | X |  |  |
| 2. Practice with WM task | X |  |  |
| 3. Motor Threshold | X |  |  |
| 4. WM task + rTMS |  | X | X |
| 5. Side Effect Checklist |  | X | X |
| 6. Visual Analog Scale |  | X | X |

**Table 2** describes the study procedures for a smaller pilot experiment, while **Figure 2** outlines the timeline and specific intra-visit timing each session.

**
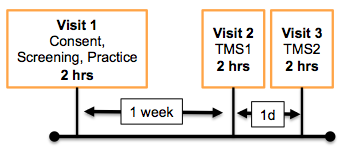
**

**Figure 2.** Timeline and timing of study procedures for the pilot study.

**Subject Screening**

Normal right-handed volunteers will be recruited from the community. At the beginning of their first visit to the rTMS laboratory, they will be screened for the study with a physical examination, portions of the MINI, and a urine test. If they pass the screening procedure, they will proceed to the rTMS lab, where they will learn and practice the memory tasks used in the study (about 1-1.5 hr), and will have a short session of rTMS (about 0.5 hr), in order to acclimate them to rTMS and to obtain right and left hand motor thresholds for future dosing of rTMS.

**WM TASKS:**

Our implementation of the WM task for MRI consists of up to 10 runs of approximately 10-minute long letter-presenting blocks. Each trial begins with the simultaneous presentation of up to 9 uppercase letters arranged in a line. In the case where fewer letters are presented, all other positions are filled with asterisks. Stimuli are presented for two seconds before the screen is cleared. After a retention interval, a probe stimulus is presented in the center of the screen for four seconds. The probe consists of a letter and a number. During this period subjects must press one of two keys indicating whether this probe was part of the memory set and reflects the appropriate response given the demands of the task. Keys (yes and no) are counterbalanced between subjects. No feedback is provided, but prior to starting the experiment subjects practice the task and feedback is provided. A three-second inter-trial interval separates trials.

In rTMS sessions, the WM tasks will be run to match the MRI version. Each block of trials will take about 9.5 minutes.

**Motor Threshold Determination**: All rTMS procedures will occur in the Noninvasive Neuromodulatory Neuroscience (N3) Lab in rooms 54211 and 54212 in the Department of Psychiatry and Behavioral Sciences, Duke Clinic South. Motor threshold (MT) is defined as the minimum magnetic flux needed to elicit a threshold EMG response in a target muscle in 5 out of 10 trials. MT is the standard in the field for determining the intensity of rTMS for each individual to reduce seizure risk. The motor evoked potentials (MEP) for the contralateral first dorsal interosseus (FDI) will be measured with EMG. The scalp region producing the largest amplitude MEP will be identified. At that scalp location, the lowest TMS intensity able to elicit 5 MEP's of ≥50µV in peak-to-peak amplitude in 10 trials at this site will be determined, using a descending method-of-limits procedure initiated by the MUSC PEST program. MT will be determined for one or both hemispheres with the muscle at rest (verified by baseline EMG). Individual MT will be used to determine the intensity of stimulation for each individual, as recommended by safety guidelines.

**MRI/FMRI PROCEDURE:** At BIAC, after obtaining a structural MRI and DTI and resting-state fMRI scans, fMRI data will be recorded from subjects while they perform the WM task. BOLD images will be acquired with a 3.0T GE MR Scanner. Stimuli will be back-projected onto a screen located at the head of the MRI bed using an LCD projector. Subjects will view the screen via a mirror system located in the head coil. Task onset will be electronically synchronized with the MRI acquisition computer. Task administration and collection of RT and accuracy data will be computer controlled. The scanning session will take about 1.5 hr.

**Application of rTMS during WM task performance**: The subject will be seated comfortably in a chair, facing a computer screen positioned in front of the subject for visual stimulus presentation. Earplugs will be worn to protect hearing. The participant's head will be held steady by a frame with a chin rest and the rTMS coil holder frame. A figure eight magnetic coil will be placed on the scalp and held in place with a coil fixing system supplied by MadVenture. This will be paired with the BrainSight tracking system which provides precise fMRI-guided rTMS coil placement for basic, translational, and clinical applications of rTMS. The BrainSight program enables to the coil to be placed at precise cortical targets (chosen from the subject’s fMRI activations from the WM task from the MRI session) on the individual’s 3-dimensionally rendered brain MRI with less than one millimeter error, and enables the research team to dynamically move the coil to account for slight head movements while the subject performs the task. Five-second trains of 5 Hz rTMS will be administered at up to 130% of the individual’s MT with the stimulating coil tangential to the scalp. Sham rTMS will be administered with a sham coil equipped with shielding to block magnetic field output but retain the auditory and some of the tactile aspects of active stimulation. rTMS administration will be controlled by an external computer and will be time-locked relative to stimulus presentation. A brief rTMS side effect rating scale and visual analog mood ratings will be administered before and after each rTMS block. There will be up to 10 blocks of 9.5 min trials per session, and each session will take about 2 hrs.

Subjects’ side-effects will be monitored closely. As indicated in the procedures section, side effects are assessed in a charted, structured interview both prior to and after the rTMS session. Subjects are instructed during the consent process and during the rTMS sessions about all known side effects. They are further instructed during consenting and additionally at the conclusion of the rTMS session to contact the principal investigator or study physician with any questions or concerns (including those that may arise after the experimental session has ended).

**5. Selection of Subjects:**

Subjects will be young or elderly adults who are appropriate research participants.

**Study Inclusion Criteria:**

1. Age restrictions
   1. Young Group: age between 18-35.
   2. Elderly Group: age between 60-80.
2. Use of effective method of birth control for women of childbearing capacity.
3. Willing to provide informed consent.

**Study Exclusion Criteria (ascertainment when appropriate):**

1. Current or recent (within the past 6 months) substance abuse or dependence, excluding nicotine and caffeine (urine test).
2. Current serious medical illness (self-report).
3. History of seizure except those therapeutically induced by ECT (childhood febrile seizures are acceptable and these subjects may be included in the study), history of epilepsy in self or first degree relatives, stroke, brain surgery, head injury, cranial metal implants, known structural brain lesion, devices that may be affected by rTMS or MRI (pacemaker, medication pump, cochlear implant, implanted brain stimulator); [TMS Adult Safety Screening (TASS) form].
4. Subjects are unable or unwilling to give informed consent.
5. Diagnosed any Axis I DSM-IV disorder (MINI, DSM-IV)
6. For subjects age > 55 years, a total scaled score < 8 on the Dementia Rating Scale-2.
7. Subjects with a clinically defined neurological disorder including, but not limited to:
   1. Any condition likely to be associated with increased intracranial pressure
   2. Space occupying brain lesion.
   3. History of stroke.
   4. Transient ischemic attack within two years.
   5. Cerebral aneurysm.
   6. Dementia.
   7. Mini Mental Status Exam (MMSE) score of <24.
   8. Parkinson’s disease.
8. Huntington’s disease.
   1. Multiple sclerosis.
9. Increased risk of seizure for any reason, including prior diagnosis of increased intracranial pressure (such as after large infarctions or trauma), or currently taking medication that lowers the seizure threshold.
10. Subjects with cochlear implants
11. Subjects not willing to tolerate the confinement associated with being in the MRI scanner.
12. Women who are pregnant or breast-feeding (urine test).

**6. Subject Recruitment & Compensation:**

Two hundred and ten volunteers will be recruited, with approximately sixty in the Young Adult (18-35 yrs. of age) cohort of Aim1 and sixty in the Older Adult (60-80 yrs.) cohort of Aim 1, including non-completers, and the remainder contributing to subsequent aims.

***Young group:*** Neurologically normal volunteers will be recruited from either 1) a subject pool maintained at the BIAC for MRI (IRB protocol Pro00010672), 2) a subject pool maintained by the Duke Laboratory of Neurogenetics (LONG) called the Duke Neurogenetics Study Database/Specimen Repository (DNS - Protocol 19095), 3) from the Egner Lab Database, 4) online advertising such as DukeList and Craigslist, or 5) via the Center for Cognitive Neuroscience Research Participation Website (http://participate.mind.duke.edu/default.asp). Male and female subjects from all racial and minority groups will be accepted.

***Elderly group:*** Neurologically normal volunteers will be recruited via 1) the Alzheimer’s Disease Prevention Registry of the Joseph & Kathleen Bryan Alzheimer’s Disease Research Center, 2) Duke Neurogenetics Study Database/Specimen Repository, 3) the CabezaLab Subject Directory, 4) the Egner Lab Database, 5) the Center for Cognitive Neuroscience Research Participation Website, 5) the Osher Lifelong Learning Institute at Duke newsletter, 6) online advertising such as DukeList, Craigslist, or Facebook or 7) from a registry maintained by the Duke Brain Imaging and Analysis Center under Pro00010672 *Screening for Participation in MRI Subject Pool,*

***Subject payment:*** $20 per hour, approximately $280.00 total for screening session (2 hrs), MRI session (2 hrs), and up to 4 rTMS sessions (10 hrs), plus a $100 bonus upon completion of all study-related visits.

**7. Consent Process:**

Section 14 questions have been completed on the e-IRB submission form.

**8. Subject’s Capacity to Give Legally Effective Consent:**

N/A: Only involves healthy volunteers

**9. Study Interventions:**

TMS and MRI procedures presented in #4 above.

**10. Risk/Benefit Assessment:**

There are no known long-term health risks to the use of magnetic resonance imaging per se when operated within FDA guidelines. However there are safety concerns posed by the strong magnetic fields used to make images. All scans conducted under this protocol meet the FDA’s guidelines for non-significant risk for static field strength, specific absorption rate (SAR), time varying magnetic fields (dB/dt), and acoustic noise.

There are no known long-term health risks to the use of rTMS per se when operated within consensus safety guidelines (Rossi et al., 2009). The Duke Medical Center Institutional Review Board recently classified two similar research applications of rTMS proposed by the PI as a “non-significant risk” (Protocols 20218 and 24349). In 2008, the FDA approved the use of high frequency rTMS in the treatment of depression. Also in 2008, an international consensus conference on safety guidelines for rTMS met, including representatives from our own labs (Drs. Lisanby and Peterchev). Their report (Rossi et al., 2009) systematically reviewed the thousands of healthy subjects and patients who have undergone rTMS in order to allow for a better assessment of relative risks. The relative infrequency of adverse events using rTMS was noted. They concluded that in the case of Class 3 studies (studies involving indirect benefit and low risk in normal subjects and patients that are expected to yield important data on brain physiology or safety, but have no immediate relevance to clinical problems), normal volunteers should be permitted to participate in rTMS research when it is likely to produce data that are of outstanding scientific or clinical value. They also concluded that this research can be performed in a non-medical setting (i.e., psychology labs, robotics labs, research institutions, etc. as opposed to a hospital or appropriately equipped outpatient clinic). The Rossi et al. consensus report went on to suggest safety guidelines based on the now rather extensive international experience with rTMS. These guidelines include the rTMS intensity and timing parameters considered safe, training, and planning for and managing emergencies. We will follow these guidelines, and have incorporated them into our screening and session procedures. The consensus safety guidelines (Rossi et al., 2009) are included as a Study Documents attachment with this IRB application.

Safety of rTMS in the elderly: The PI of the present study (Dr. Appelbaum) previously ran a study involving thirty-five healthy elderly subjects under an approved Duke IRB protocol without incident. As the present study is based on our successful work in that protocol, the paradigm is virtually the same, including the design of the rTMS sessions, the use of the WM task, and the use of 7 second trains of 5 Hz rTMS during task performance. All of the elderly subjects completed the protocol quite smoothly: no adverse events occurred, and subjects reported mild side effects (like headache) at the same frequency as younger volunteers had.

TMS has been used with elderly participants in a number of clinical studies. Application of rTMS has aided recovery in post stroke patients (Brown et al., 2006; Kim et al., 2006; Jorge et al., 2004) and in older adults suffering from Parkinson's disease (Fregni et al., 2004; Boggio et al., 2005). It has been used in a number of investigations of depression, with mixed results (Manes et al., 2001; Moser et al., 2002; Mosimann et al., 2004; Nahas et al., 2004). Two of the depression studies reported increases in cognitive performance with rTMS (Moser et al., 2002; Boggio et al., 2005). There have been no reports of serious adverse events with the use of rTMS in elderly subjects in all of the clinical studies reported to date. No seizures have ever been reported in normal subjects, including the elderly, when high frequency rTMS was administered within the established safety guidelines. Three studies of depression in the elderly specifically looked at issues of safety and side effects (Manes et al., 2001; Mosimann et al., 2004; Nahas et al., 2004). In Manes et al., 10 depressed elderly patients received 20 2-second trains of 20 Hz rTMS for 20 minutes over five days. In the 50 sessions, there were four mild headaches, five cases of scalp pain near the point of stimulation, and one case of anxiety. In Mosimann et al., 25 elderly patients received 40 2-second trains of 20 Hz rTMS for 20 minutes over ten days, 15 with active rTMS and 9 with sham. About half of the active and the sham groups reported mild side effects, including mild headaches; no serious side effects were reported. In Nahas et al., 18 patients received 40 8-second trains of 5 Hz rTMS for 20 minutes over 15 sessions in three weeks. The only side effects reported were three cases of mild headaches.

Participation is voluntary, and there will be no pressure or time constraints regarding the decision to participate. There are no benefits to the participants except for the monetary reward or compensation, as well as the good will of helping the progress of scientific research. The information learned from this study may aid our understanding how the brain holds information on-line (WM) and changes in the brain's memory function due to age. This knowledge may lead us towards future interventions to improve aspects of cognitive performance in the elderly.

MRI Adverse Events Plan:

An MRI procedure is considered to be “minimal risk” according to federal definitions. To date, no after effects have been revealed and the FDA has classified the MR procedure as possessing a "non significant risk" for the subject of study. To minimize risks, all subjects will be screened for metallic devices, implants and other contraindications to scanning. Women of child-bearing capacity will be excluded for pregnancy using a pregnancy test prior to scanning. Those unlikely to tolerate the sense of confinement during scanning will also be excluded.

Adequate safety monitoring and observation during scanning will be provided, as will measures to enhance the subject’s physical and emotional comfort during the scan. It is possible that some subjects might experience minor distress by the confined and noisy conditions in the scanner. This possibility will be minimized by earplugs and headphones, and experienced technicians who will monitor all subjects for distress. In the event that a subject becomes anxious during a scan, the study will be halted. Subjects will be able to communicate with the investigators at all times using the intercom system should they wish to request that a study be terminated or have concerns or questions during the procedure. The subject is in full view of the operator at all times.

The probability of an incidental finding that might lead to the diagnosis of an unknown abnormality is greater than zero. All subjects who undergo MR-related procedures will be alerted to this possibility during the consent process. In that event, subjects or their designated physician will be provided copies of their anatomical scans and advised to seek further evaluation if they have concerns.

TMS Adverse Events Plan:

Seizure is a theoretical risk with rTMS. In the Rossi et al. report it was stated that “The occurrence of seizures has been extremely rare, with most of the few new cases receiving rTMS protocols exceeding previous guidelines, often in patients under treatment with drugs which potentially lowered the seizure threshold.” As Rossi et al. delineate, “rare” means that 16 cases (out of tens of thousands of rTMS sessions over the last two decades) of seizure related to rTMS have been reported. Eight occurred before safety parameters were established in 1997. Of the other eight reports, six occurred either when the safe rTMS parameters were exceeded or other safety guidelines ignored, and the actual occurrence of a seizure has been questioned in the other two (i.e., convulsive syncope or pseudoseizure may have occurred). In a workshop convened by the National Institute for Neurological Disorders and Stroke (NINDS) in 1996 (Duke investigators, Dr. Luber, and Dr. Lisanby were participants), researchers in the field agreed upon a set of *rTMS consensus safety guideline*s, including recommended stimulation parameters and contra-indications (Wassermann, 1998), and these consensus guidelines have been recently updated (Rossi et al., 2009). Widespread adherence to the 1996 guidelines has resulted in the virtual elimination of inadvertent seizures in rTMS studies (Rossi et al., 2009). The levels of stimulation used in this protocol are well within safety guidelines (Rossi et al., 2009; Wassermann et al. 1998).

We will screen subjects for known risk factors for seizure with rTMS (medical screening and medical history). Personnel who administer rTMS are trained to recognize a potential seizure event and to act as “first responders” in order to administer appropriate initial care. All study personnel have undergone Basic Life Saving training, and seizure-specific training. The major physical signs the study personnel will look out for in detecting a potential seizure include chewing movements, convulsions/tremor/shaking, difficulty talking, a blank stare, eyes rolling up, and profuse sweating. If any of these signs are observed, study personnel will stop the research procedure and inquire whether the subject feels okay. If the subject is unresponsive (and therefore likely experiencing a seizure), first-aid will be supplied. The first-aid response consists of making sure the subject is physically safe for the duration of the seizure. This involves moving the subjects out of the rTMS chair and onto the floor lying down on his or her left side. The subject will be kept lying down on his or her left side, while the staff call emergency medical help, via a 911 call. Resources available in the laboratory include a first-aid kit and immediate phone access. A seizure constitutes a reportable adverse event, and will thus be immediately reported to the IRB via the Safety Events Form mechanism.

The most commonly reported side effect of rTMS is headache. This headache is typically of a muscle-tension type. It usually develops during or immediately after the stimulation and may last for minutes to hours following the end of the stimulation. It is typically limited to the day of stimulation, and usually responds promptly to single doses of over the counter pain medications. Neck pain or scalp pain may also occur. Both are usually managed easily with over-the-counter analgesics. Participants may take Advil or Tylenol to reduce discomfort. Tylenol or Advil will be used according to recommended dosage, ensuring that the participant has no allergies and has not taken other NSAIDS or acetaminophen. Liver damage can occur when more than 4,000 mg of acetaminophen are takin in 24 hours or when taken with alcohol. Ibuprofen can cause stomach bleeding in certain individuals or when not adhering to dosage recommendations. Once the Advil or Tylenol has had time to take effect, we will resume stimulation. If there is still discomfort, topical lidocaine cream may be applied to the area of the scalp where stimulation occurs. Topical lidocaine has been shown to relieve discomfort resulting from rTMS for some people. Topical lidocaine should not be used on large areas or on cut, irritated or swollen skin. Twenty minutes must be allowed for the lidocaine cream to take effect. If implemented, Tylenol, Advil, and lidocaine cream will be used as directed.

As noted in Rossi et al. (2009), Loo and colleagues reported mild and transient changes in auditory threshold in two depressed patients following a 2-4 week rTMS course of rTMS (Loo et al., 2001). Cases of tinnitus have been reported after rTMS treatments. In addition, recently in a study investigating the effects of rTMS on symptoms of depression, a patient experienced moderate to severe tinnitus after an rTMS session in which earplugs were not used. Rossi et al. recommended that hearing protection always should be worn during rTMS application, and that individuals with cochlear implants not receive rTMS. In the current study, earplugs will be worn by all subjects during rTMS procedures. Individuals with cochlear implants will be excluded from participation.

Risks to the unborn children of pregnant women receiving MRI and rTMS are unknown. Pregnant women will be excluded as per IRB policy. Female subjects are tested once with a urine pregnancy test prior to their first MRI session as per IRB-approved BIAC policy. These female subjects agree not to become pregnant while remaining within the subject pool, and to notify the experimenter or subject coordinator if they become pregnant. If sexually active, the subject must agree to use appropriate contraceptive measures for the duration of the study. Medically acceptable contraceptives include: (1) surgical sterilization (such as a tubal ligation or hysterectomy), (2) approved hormonal contraceptives (such as birth control pills, patches, implants or injections), (3) barrier methods (such as a condom or diaphragm) used with a spermicide, or (4) an intrauterine device (IUD). Contraceptive measures such as Plan B (TM), sold for emergency use after unprotected sex, are not acceptable methods for routine use. If the subject has any uncertainty about whether they could be pregnant, another urine pregnancy test will be performed before they can participate in this protocol. The person(s) who will perform the urine pregnancy test will have successfully completed training as directed by the Chair of Obstetric and Gynecology of the Duke University School of Medicine. The urine pregnancy test kits used for this research study will be those commercially available test kit specified by the Chair of Obstetric and Gynecology and in routine use at DUHS.

**11. Costs to the Subject:**

There is no cost for subjects to participate in this study.

**12. Data Analysis & Statistical Considerations:**

**fMRI data*:*** All image pre-processing will be implemented using the SPM12 program (http://www.fil.ion.ucl.ac.uk/spm/software/**spm12**). Event-related fMRI data will be extracted by time-series modeling using rectangular time windows for the memory encoding and probe phases of the task, one modeling a brief (400 ms) neural response at the beginning of the task phase, and another modeling a neural response lasting for the entire 3000 ms phase. A single rectangular function of 7000 ms will be used for the retention phase. Group data will then be analyzed using ordinal trends model (OrT: a newly-developed multivariate covariance analysis). Any fMRI network related to performance in the WM task from previous fMRI research using the WM task in young and elderly subjects will be prospectively applied to the fMRI data of the subjects. Specifically, the imaging data from the pre and post MRI sessions will be log transformed and subtracted from each other at each site in each subject. The global mean across sites will be subtracted from each log-transformed regional value, producing regional values normalized to each subject’s global mean activity. In a second normalization, the mean for each site across subjects will be subtracted from each regional value. The result of these two normalizations is a subject x region matrix with the mean activity within and across subjects removed. This will be cross-correlated with the patterns previously found in order to quantify pattern expression for individual subjects in the present group. These individual subject pattern expressions will be entered into regressions predicting WM performance in order to verify that the patterns predict performance in new group. In addition, pattern expression will be used to predict rTMS effects on WM performance (for example, differences in RT with active and sham rTMS).

**TMS data:** Mixed model ANOVAs for the two groups with repeated-measures factors of Site (2 scalp locations), Study Set Size (three and six), and rTMS (Active and Sham) will be performed separately on the median RT and accuracy data. Bn at an alpha of 0.05. In our previous studies using the WM task with active and sham rTMS over multiple sites, letter set sizes, and task phases, groups of 21 (Luber et al., 2007) 15 (Luber et al., 2008) and 26 (Luber et al., 2013) were sufficient to produce significant effects at the 0.05 level.

**13. Data & Safety Monitoring:**

The subjects will be fully informed of the nature of the study requirements prior to enrollment and periodically throughout the study. The subject’s wellbeing will be continuously monitored by the experimenter, and the Principal Investigator will report all serious adverse events in an expedited manner to the Duke University Health System (DUHS) Institutional Review Board (IRB) office and all applicable regulatory authorities in accordance with the Center’s standard operating procedures.

The study monitor will be Dr. Greg Appelbaum. Dr. Appelbaum will ensure the quality of the study and establish that each co-investigator is complying with the investigational plan and IRB regulations. Monitoring of this protocol is simplified by the fact that this study involves a small number of investigators and a single facility in which the study is being conducted. Drs. Bruce Luber and Sarah H. Lisanby will be involved as Outside Consultants, as both have transitioned from Duke University Medical Center to positions at the NIMH. Their roles will not require the sharing of PHI, but will guide study design decisions for the lifetime of the study given their collective expertise in TMS.

Throughout the investigation, the monitor will ensure that the facilities being used continue to be acceptable for the purposes of the study, that the investigational plan is being followed, that any changes to the protocol have received IRB approval and have been reported to the sponsor, that accurate, complete, and current records are maintained, that accurate, complete, and timely reports are made to the IRB. This will be accomplished through quarterly meetings during which the status of the protocol, investigators, and IRB compliance are reviewed. The monitor will review each research chart for completeness and accuracy. He will confirm that inclusion and exclusion criteria have been met for each subject enrolled, and compliance with all other aspects of the investigational plan are met.

**14. Privacy, Data Storage & Confidentiality:**

Section 12 of the e-IRB submission form have been completed.

**References**

Boggio PS, Fregni F, Bermpohl F, Mansur CG, Rosa M, Rumi DO, Barbosa ER, Odebrecht Rosa M, Pascual-Leone A, Rigonatti SP, Marcolin MA, Araujo Silva MT (2005) Effect of repetitive TMS and fluoxetine on cognitive function in patients with Parkinson's disease and concurrent depression. Mov Disord 20:1178-1184.

Carp J, Gmeindl L, Reuter-Lorenz PA (2010) Age differences in the neural representation of working memory revealed by multi-voxel pattern analysis. Frontiers in human neuroscience 4:217.

Fregni F, Santos CM, Myczkowski ML, Rigolino R, Gallucci-Neto J, Barbosa ER, Valente KD, Pascual-Leone A, Marcolin MA (2004) Repetitive transcranial magnetic stimulation is as effective as fluoxetine in the treatment of depression in patients with Parkinson's disease. J Neurol Neurosurg Psychiatry 75:1171-1174.

Hambrick DZ, Engle RW (2002) Effects of domain knowledge, working memory capacity, and age on cognitive performance: an investigation of the knowledge-is-power hypothesis. Cogn Psychol 44:339-387.

Harada CN, Natelson Love MC, Triebel KL (2013) Normal cognitive aging. Clin Geriatr Med 29:737-752.

Herwig U, Satrapi P, Schonfeldt-Lecuona C (2003) Using the international 10-20 EEG system for positioning of transcranial magnetic stimulation. Brain Topogr 16:95-99.

Loo C, Sachdev P, Elsayed H, McDarmont B, Mitchell P, Wilkinson M, Parker G, Gandevia S (2001) Effects of a 2- to 4-week course of repetitive transcranial magnetic stimulation (rTMS) on neuropsychologic functioning, electroencephalogram, and auditory threshold in depressed patients. Biol Psychiatry 49:615-623.

Luber B (2014) Neuroenhancement by noninvasive brain stimulation is not a net zero-sum proposition. Front Syst Neurosci 8:127.

Luber B, McClintock SM, Lisanby SH (2013) Applications of transcranial magnetic stimulation and magnetic seizure therapy in the study and treatment of disorders related to cerebral aging. Dialogues in clinical neuroscience 15:87-98.

Luber B, Kinnunen LH, Rakitin BC, Ellsasser R, Stern Y, Lisanby SH (2007) Facilitation of performance in a working memory task with rTMS stimulation of the precuneus: frequency- and time-dependent effects. Brain Res 1128:120-129.

Luber B, Stanford AD, Bulow P, Nguyen T, Rakitin BC, Habeck C, Basner R, Stern Y, Lisanby SH (2008) Remediation of sleep-deprivation-induced working memory impairment with fMRI-guided transcranial magnetic stimulation. Cereb Cortex 18:2077-2085.

Manes F, Jorge R, Morcuende M, Yamada T, Paradiso S, Robinson RG (2001) A controlled study of repetitive transcranial magnetic stimulation as a treatment of depression in the elderly. Int Psychogeriatr 13:225-231.

Moser DJ, Jorge RE, Manes F, Paradiso S, Benjamin ML, Robinson RG (2002) Improved executive functioning following repetitive transcranial magnetic stimulation. Neurology 58:1288-1290.

Mosimann UP, Schmitt W, Greenberg BD, Kosel M, Muri RM, Berkhoff M, Hess CW, Fisch HU, Schlaepfer TE (2004) Repetitive transcranial magnetic stimulation: a putative add-on treatment for major depression in elderly patients. Psychiatry research 126:123-133.

Nahas Z, Li X, Kozel FA, Mirzki D, Memon M, Miller K, Yamanaka K, Anderson B, Chae JH, Bohning DE, Mintzer J, George MS (2004) Safety and benefits of distance-adjusted prefrontal transcranial magnetic stimulation in depressed patients 55-75 years of age: a pilot study. Depress Anxiety 19:249-256.

Rossi S, Hallett M, Rossini PM, Pascual-Leone A, Safety of TMSCG (2009) Safety, ethical considerations, and application guidelines for the use of transcranial magnetic stimulation in clinical practice and research. Clinical neurophysiology : official journal of the International Federation of Clinical Neurophysiology 120:2008-2039.

Rozas AX, Juncos-Rabadan O, Gonzalez MS (2008) Processing speed, inhibitory control, and working memory: three important factors to account for age-related cognitive decline. Int J Aging Hum Dev 66:115-130.

Wassermann EM (1998) Risk and safety of repetitive transcranial magnetic stimulation: report and suggested guidelines from the International Workshop on the Safety of Repetitive Transcranial Magnetic Stimulation, June 5-7, 1996. Electroencephalogr Clin Neurophysiol 108:1-16.

Ziemann U (2004) TMS induced plasticity in human cortex. Rev Neurosci 15:253-266.

Zimprich D, Kurtz T (2013) Individual differences and predictors of forgetting in old age: the role of processing speed and working memory. Neuropsychology, development, and cognition Section B, Aging, neuropsychology and cognition 20:195-219.
