# Supplementary material for: Online repetitive transcranial magnetic stimulation during working memory in younger and older adults: A randomized within-subject comparison
Source: PLoS One. 2019 Mar 22;14(3):e0213707. doi: 10.1371/journal.pone.0213707 (PMC6430375; doi:10.1371/journal.pone.0213707)
Supplement: S3 File — (DOC) [file pone.0213707.s003.doc]

| SUBJECT INITIALS  🞏🞏🞏 | DATE OF VISIT  🞏🞏/🞏🞏/🞏🞏  MM DD YY | **^VISIT^**  **Screening** | |  |
| --- | --- | --- | --- | --- |
|  | | | |  |
| **Check one→** | | **Yes*** | **No** | **N/A** |
| Have you ever… | |  |  |  |
| …had an adverse reaction to rTMS? | |  |  |  |
| …had a seizure? | |  |  |  |
| …had an EEG? | |  |  |  |
| …had a stroke? | |  |  |  |
| …had a head injury (including neurosurgery)? | |  |  |  |
| Do you have any metal in your head (outside of the mouth) such as shrapnel, surgical clips, or fragments from welding or metalwork? | |  |  |  |
| Do you have any implanted devices such as cardiac pacemakers, medical pumps, or intracardiac lines? | |  |  |  |
| Do you suffer from frequent or severe headaches? | |  |  |  |
| Have you ever had any other brain-related condition? | |  |  |  |
| Have you ever had any illness that caused brain injury? | |  |  |  |
| Are you taking any medications? | |  |  |  |
| If you are woman of childbearing age, are you sexually active, and if so, are you *not using* a reliable method of birth control? | |  |  |  |
| Does anyone in your family have epilepsy? | |  |  |  |
| Do you need further explanation of rTMS and its associated risks? | |  |  |  |
| ***Explain any ‘YES’ answers in the comment section below.** | |  |  |  |
| Comments: | | | |  |
|  | | | |  |
|  | | | |  |
|  | | | |  |
|  | | | |  |
