# Supplementary material for: Online repetitive transcranial magnetic stimulation during working memory in younger and older adults: A randomized within-subject comparison
Source: PLoS One. 2019 Mar 22;14(3):e0213707. doi: 10.1371/journal.pone.0213707 (PMC6430375; doi:10.1371/journal.pone.0213707)
Supplement: S4 File — (DOCX) [file pone.0213707.s004.docx]

**Study Inclusion Criteria:**

1. Age restrictions
   1. Young Group: age between 18-35.
   2. Elderly Group: age between 60-80.
2. Use of effective method of birth control for women of childbearing capacity.
3. Willing to provide informed consent.

**Study Exclusion Criteria (ascertainment when appropriate):**

1. Current or recent (within the past 6 months) substance abuse or dependence, excluding nicotine and caffeine (urine test).
2. Current serious medical illness (self-report).
3. History of seizure except those therapeutically induced by ECT (childhood febrile seizures are acceptable and these subjects may be included in the study), history of epilepsy in self or first degree relatives, stroke, brain surgery, head injury, cranial metal implants, known structural brain lesion, devices that may be affected by rTMS or MRI (pacemaker, medication pump, cochlear implant, implanted brain stimulator); [TMS Adult Safety Screening (TASS) form]. (See S3 File)
4. Subjects are unable or unwilling to give informed consent.
5. Diagnosed any Axis I DSM-IV disorder (MINI, DSM-IV)
6. For subjects age > 55 years, a total scaled score < 8 on the Dementia Rating Scale-2.
7. Subjects with a clinically defined neurological disorder including, but not limited to:
   1. Any condition likely to be associated with increased intracranial pressure
   2. Space occupying brain lesion.
   3. History of stroke.
   4. Transient ischemic attack within two years.
   5. Cerebral aneurysm.
   6. Dementia.
   7. Mini Mental Status Exam (MMSE) score of <24.
   8. Parkinson’s disease.
8. Huntington’s disease.
   1. Multiple sclerosis.
9. Increased risk of seizure for any reason, including prior diagnosis of increased intracranial pressure (such as after large infarctions or trauma), or currently taking medication that lowers the seizure threshold.
10. Subjects with cochlear implants
11. Subjects not willing to tolerate the confinement associated with being in the MRI scanner.
12. Women who are pregnant or breast-feeding (urine test).
