# Supplementary material for: Online repetitive transcranial magnetic stimulation during working memory in younger and older adults: A randomized within-subject comparison
Source: PLoS One. 2019 Mar 22;14(3):e0213707. doi: 10.1371/journal.pone.0213707 (PMC6430375; doi:10.1371/journal.pone.0213707)
Supplement: S5 File — (PPTX) [file pone.0213707.s005.pptx]

## Slide 1
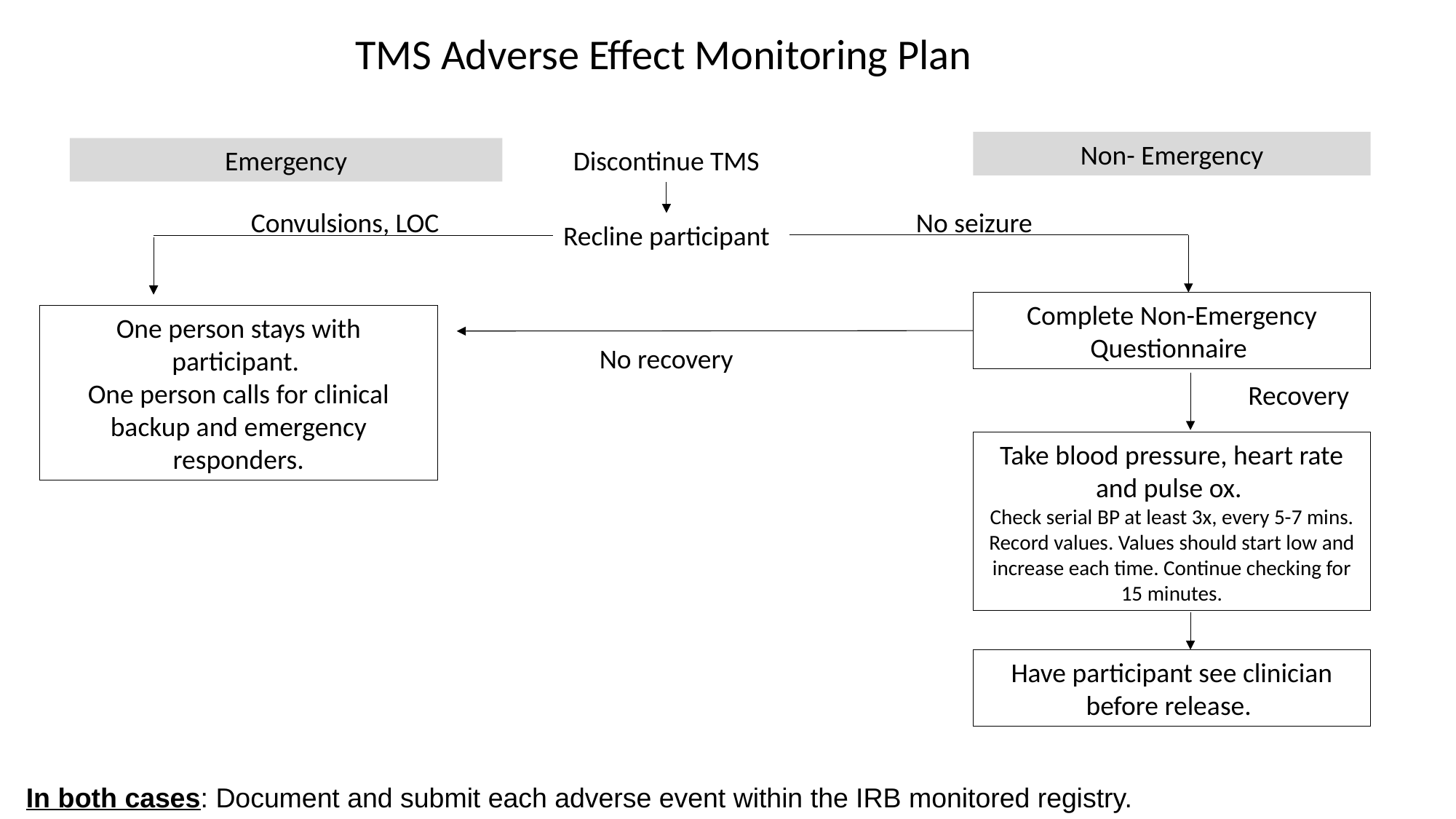

TMS Adverse Effect Monitoring Plan
Non- Emergency
Emergency
Discontinue TMS
Convulsions, LOC
No seizure
Recline participant
Complete Non-Emergency Questionnaire
One person stays with participant.
One person calls for clinical backup and emergency responders.
No recovery
Recovery
Take blood pressure, heart rate and pulse ox.
Check serial BP at least 3x, every 5-7 mins.
Record values. Values should start low and increase each time. Continue checking for 15 minutes.
Have participant see clinician before release.
In both cases: Document and submit each adverse event within the IRB monitored registry.

## Slide 2
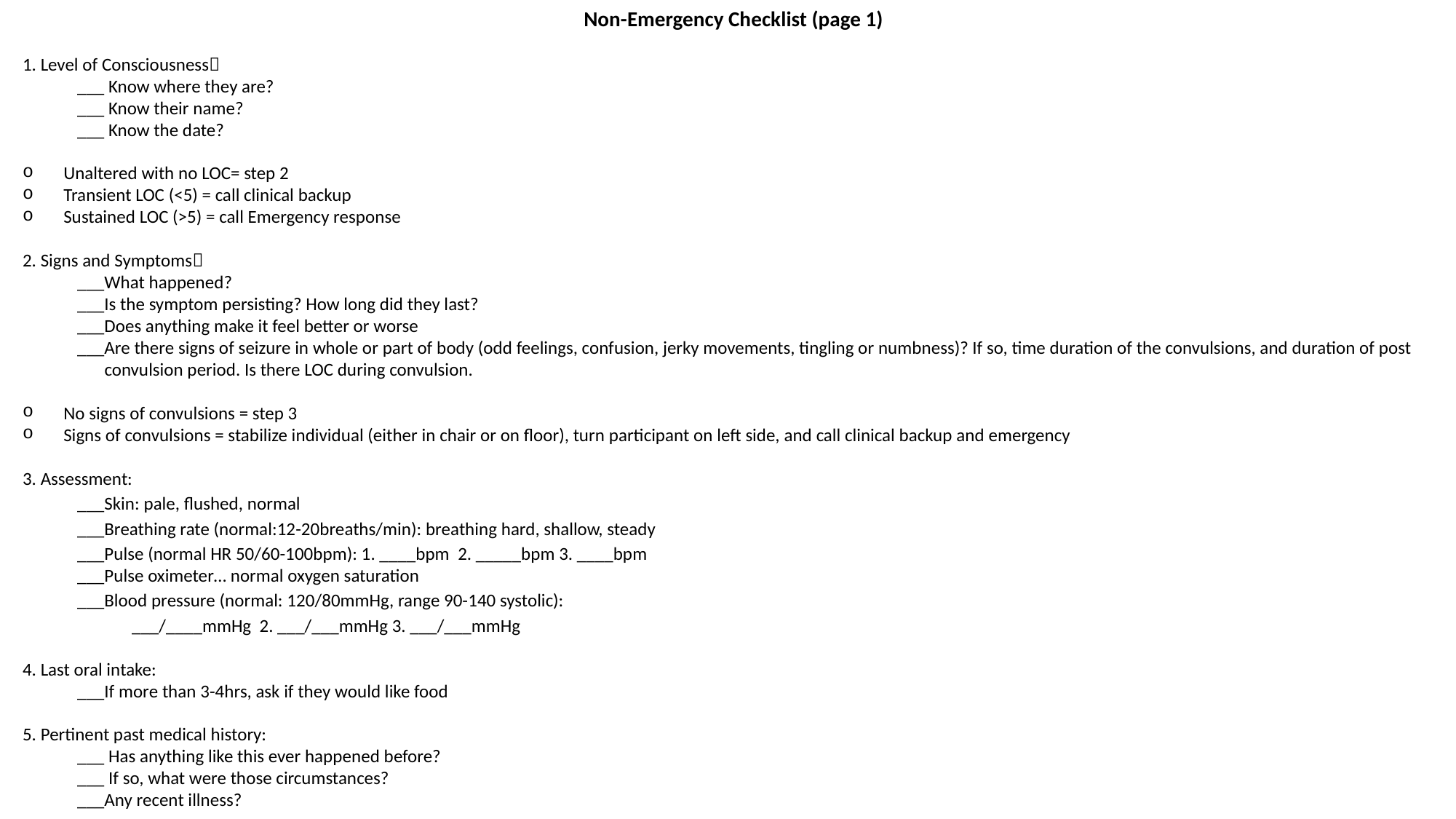

Non-Emergency Checklist (page 1)
1. Level of Consciousness
___ Know where they are?
___ Know their name?
___ Know the date?
Unaltered with no LOC= step 2
Transient LOC (<5) = call clinical backup
Sustained LOC (>5) = call Emergency response
2. Signs and Symptoms
___What happened?
___Is the symptom persisting? How long did they last?
___Does anything make it feel better or worse
___Are there signs of seizure in whole or part of body (odd feelings, confusion, jerky movements, tingling or numbness)? If so, time duration of the convulsions, and duration of post convulsion period. Is there LOC during convulsion.
No signs of convulsions = step 3
Signs of convulsions = stabilize individual (either in chair or on floor), turn participant on left side, and call clinical backup and emergency
3. Assessment:
___Skin: pale, flushed, normal
___Breathing rate (normal:12-20breaths/min): breathing hard, shallow, steady
___Pulse (normal HR 50/60-100bpm): 1. ____bpm 2. _____bpm 3. ____bpm
___Pulse oximeter… normal oxygen saturation
___Blood pressure (normal: 120/80mmHg, range 90-140 systolic):
	___/____mmHg 2. ___/___mmHg 3. ___/___mmHg
4. Last oral intake:
___If more than 3-4hrs, ask if they would like food
5. Pertinent past medical history:
___ Has anything like this ever happened before?
___ If so, what were those circumstances?
___Any recent illness?

## Slide 3
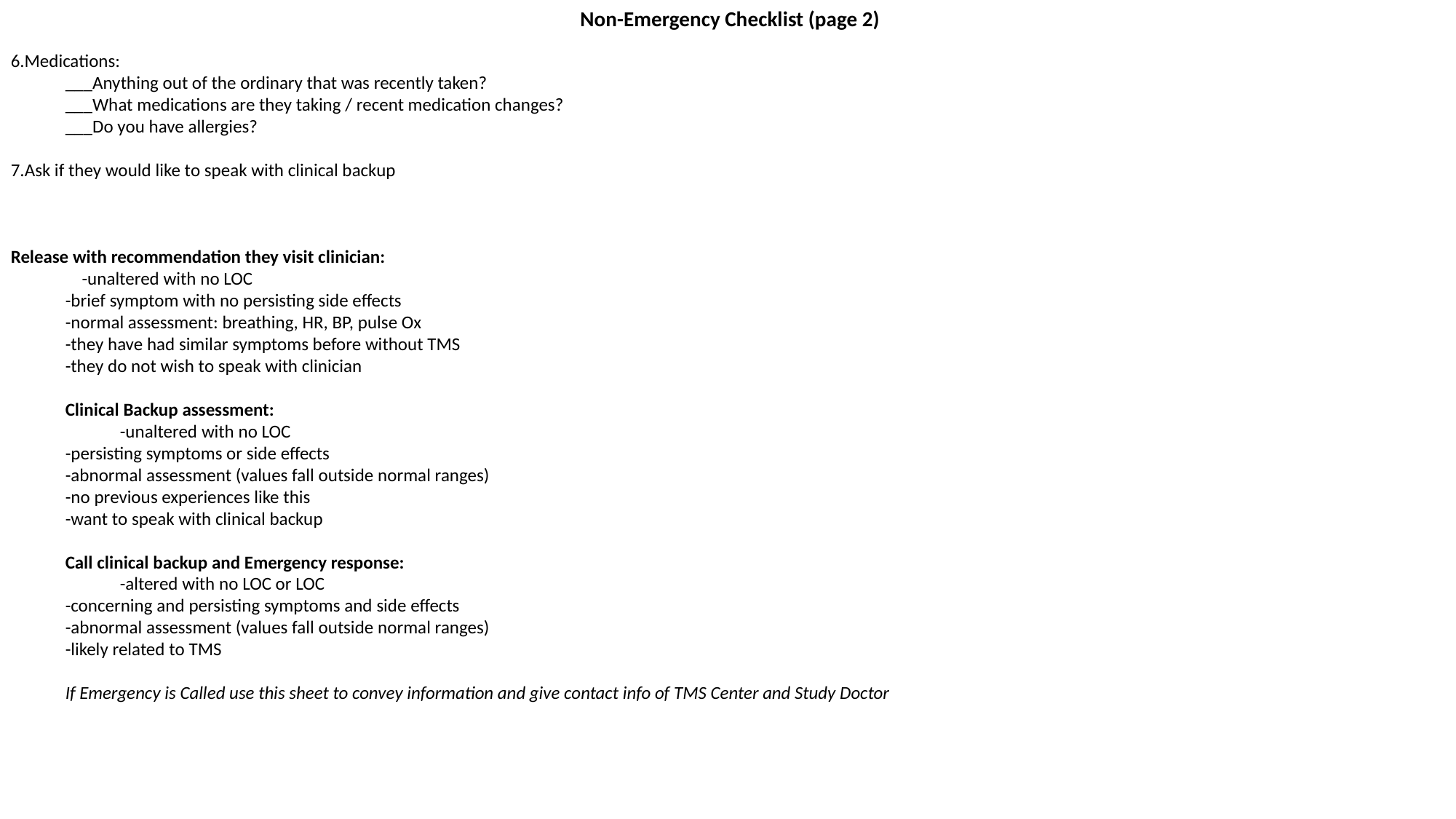

Non-Emergency Checklist (page 2)
6.Medications:
___Anything out of the ordinary that was recently taken?
___What medications are they taking / recent medication changes?
___Do you have allergies?
7.Ask if they would like to speak with clinical backup
Release with recommendation they visit clinician:
 -unaltered with no LOC
-brief symptom with no persisting side effects
-normal assessment: breathing, HR, BP, pulse Ox
-they have had similar symptoms before without TMS
-they do not wish to speak with clinician
Clinical Backup assessment:
	-unaltered with no LOC
-persisting symptoms or side effects
-abnormal assessment (values fall outside normal ranges)
-no previous experiences like this
-want to speak with clinical backup
Call clinical backup and Emergency response:
	-altered with no LOC or LOC
-concerning and persisting symptoms and side effects
-abnormal assessment (values fall outside normal ranges)
-likely related to TMS
If Emergency is Called use this sheet to convey information and give contact info of TMS Center and Study Doctor
